# Amazing Types, Properties, and Applications of Fibres in Construction Materials

**DOI:** 10.3390/ma12162513

**Published:** 2019-08-07

**Authors:** Abbas Mohajerani, Siu-Qun Hui, Mehdi Mirzababaei, Arul Arulrajah, Suksun Horpibulsuk, Aeslina Abdul Kadir, Md Tareq Rahman, Farshid Maghool

**Affiliations:** 1 Civil and Infrastructure Engineering, School of Engineering, RMIT University, Melbourne 3000, Australia; 2 School of Engineering and Technology, CQUniversity, Melbourne 3000, Australia; 3 Department of Civil and Construction Engineering, Swinburne University of Technology, Hawthorn 3122, Australia; 4 School of Civil Engineering, Suranaree University of Technology, Nakhon Ratchasima 30000, Thailand,; 5 Faculty of Civil and Environmental Engineering, Universiti Tun Hussein Onn Malaysia (UTHM), Batu Pahat 86400, Malaysia

**Keywords:** natural fibres, synthetic fibres, basalt fibres, waste fibres, fibre reinforcement, construction materials. concrete, asphalt, soil, composite materials, environmental impacts

## Abstract

Fibres have been used in construction materials for a very long time. Through previous research and investigations, the use of natural and synthetic fibres have shown promising results, as their presence has demonstrated significant benefits in terms of the overall physical and mechanical properties of the composite material. When comparing fibre reinforcement to traditional reinforcement, the ratio of fibre required is significantly less, making fibre reinforcement both energy and economically efficient. More recently, waste fibres have been studied for their potential as reinforcement in construction materials. The build-up of waste materials all around the world is a known issue, as landfill space is limited, and the incineration process requires considerable energy and produces unwanted emissions. The utilisation of waste fibres in construction materials can alleviate these issues and promote environmentally friendly and sustainable solutions that work in the industry. This study reviews the types, properties, and applications of different fibres used in a wide range of materials in the construction industry, including concrete, asphalt concrete, soil, earth materials, blocks and bricks, composites, and other applications.

## 1. Introduction

All around the world, people use materials to build and construct structural and nonstructural products in a diverse range of industries [1]. Materials are required to form bricks, and bricks can be used to build houses, which are a necessity for a community. For thousands of years, earth materials have been used in construction, and continue to be used in present times [2]. Naturally, the types of construction material vary around the world, as economies and readily available materials differ.

Globally, fibres have been used in construction materials for a very long time. Moreover, there has been an extended history of composite materials, in which fibres have been used as reinforcement to enhance the properties of the materials [3]. Dating back to the Egyptian period, natural fibres like straw and horsehair were incorporated in the formation of mud bricks [4]. Straw was a common material used in early Japanese and Chinese construction, purposed as reinforcement for structural components of houses [4]. As of the late 1800s, the United States adopted straw material as a main component of bearing walls [4].

In particular, coconut (coir), sisal, palm, jute, flax, straw, bamboo, and cane have a long history as they are readily available and can be procured from the natural environment [4]. With natural fibres, the condition of the surrounding environment at the time of harvest will influence their physical properties and mechanical properties [5]. This includes the condition of the soil, the process used for the extraction, any treatment undertaken, air humidity, temperature, and more [5].

Concrete is a common element used in the construction industry. For roughly 50 years, fibres have been added to concrete applications in the construction and civil industry [6]. More recently, fibre-reinforced polymers have been used in the construction industry for over two decades, demonstrating positive benefits such as increased strength [7] and being lightweight and resistant to corrosion.

Each construction material has properties in which it is poor, and properties in which it excels. For instance, a key property for which concrete is acknowledged is its high compressive strength. Conversely it possesses low tensile strength [8,9]. Likewise, soil is typically poor in tensile and shear strength [4]. Fibres are a valuable material additive for construction materials [10]. Specific fibres can be selected and introduced into these construction materials to improve the properties in which the parent material is deprived, making the resultant material well-rounded. For synthetic fibres, different production methods and raw material compositions will lead to different mechanical properties of the fibres [9].

Fibres have different characteristics and come in a range of shapes and sizes. If implemented, the fibre type will need to be carefully considered and chosen, as some fibres, such as glass, basalt, and recycled polyethylene terephthalate fibres, can be degraded in alkaline environments [7,9,11].

Fibres can reduce the total cost of the construction, as they can be used as reinforcement, replacing the traditional and energy consuming methods of wired mesh and steel reinforcement bars [9,12]. As a result, there will be less labour cost, less maintenance cost, time saved from the construction works, and lower build cost. Energy will also be saved, as the quantity of fibre used is generally significantly less than the volume of raw material required to manufacture the traditional reinforcement.

Overall, there are many benefits of incorporating fibres into construction materials. There is also the potential for incorporating waste fibres into construction materials, which utilises waste materials, reduces the volume of landfill, saves energy, is environmentally friendly, and promotes sustainable solutions towards work in the industry.

### 1.1. Objective

The objective of this study is to review the types, properties, and applications of different fibres used in a wide range of construction materials including normal concrete, asphalt concrete, soil, earth materials, blocks and bricks, composites, and other applications. Furthermore, the advantages, disadvantages, and limitations, as well as the environmental impacts, of fibres are reviewed and discussed.

In each section of the application of fibres in construction materials, common issues that arise in the construction material are discussed. The results of previous research and studies that successfully attempted to utilise different fibres to overcome these issues are then reviewed.

**Figure 1:**
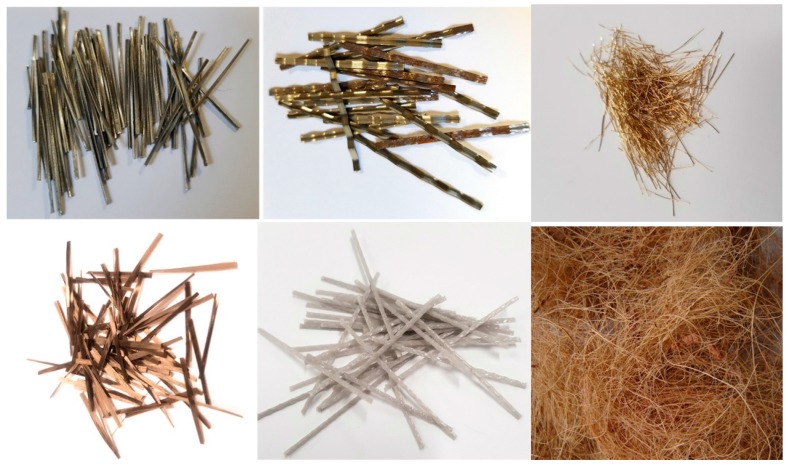
Samples of steel and copper fibres (top row), and basalt, plastic, and coconut fibres (bottom row).

Finally, this paper summarises the different types of fibres, their properties, applications, and advantages, and disadvantages when used in the construction industry. Furthermore, some recommendations for further study are suggested. Samples of some of the fibres reviewed in this study are shown in Figure 1.

### 1.2. Method of Study

Literature reviews, case studies, and various papers addressing fibres and fibres in different construction materials were collected. The publication date of the selected papers ranged from 1999 to 2018. Overall, a total of 55 different papers were studied for this review.

## 2. Types and Properties of Fibres

Fibres are typically classified as either natural fibres or synthetic fibres. Natural fibres are found in the environment, and are typically extracted from the exterior of plants, trees, and straw [4]. Natural fibres have a longer history when compared to synthetic fibres. Synthetic fibres are manufactured fibres that are formed for a purpose [4]. A summary table of fibres and their properties from various references is included in Table A1 (Appendix A).

### 2.1. Natural Fibres

Natural fibres include coconut (coir), sisal, palm, jute, flax, straw, bamboo, cane, and many more [4,5]. Furthermore, natural fibres can be distinguished into various subcategories, such as cereal straw, wood fibres, basalt fibres, palm tree fibres, and leaf fibres [2,13]. With natural fibres, the condition of the surrounding environment at the time of harvest will influence the physical and mechanical properties [5]. This includes the condition of the soil, the process used for extraction, any treatment undertaken, air humidity, temperature, and more [5].

The use of natural fibres has a long history. Different natural fibres are found all around the world and are readily available as they grow naturally in the environment. They are readily available, abundant, require little to no energy to produce, can be extracted from waste material, are economical and low in cost, and can reduce the environmental impact when used in the construction industry [2,4]. The use of natural fibres can also support the use of sustainable materials [4]. However, it should be noted that the main disadvantage to these fibres is that, typically, they have poor durability and because they are natural, they may degrade by natural means over time [9,14]. Another disadvantage of plant fibres is that they are hydrophilic [15].

There have been recent studies to try to improve the durability of the natural fibres for composite materials [14]. These include chemical treatment, coating, and substituting or blocking the hydroxyl group of the organic natural fibres. Nevertheless, further research is required to determine if the improvement in terms of durability is long-term or just temporary [14]. It has been stated that these methods are quite costly and could be hazardous as leeching and exposure to biological, chemical, thermal, and UV-related degradation have not been investigated in detail [14].

A number of natural fibres are agricultural waste products that either get dumped in landfills or burnt [16]. The natural fibre-reinforced composites show the potential for recyclability and biodegradability [17]. Natural fibre-reinforced composites can have improved properties, such as improved strength, and control shrinkage and the formation of cracks. When natural fibres are utilised, waste materials in landfills can be reduced, emissions can be prevented, and the energy used in the burning procedure can be saved.

Easily renewable materials with optimal properties and ecology have been a trend of study in the civil engineering industry [18]. Two main ways to achieve sustainability are to utilise natural fibres and industrial waste materials [18]. Natural materials tend to have good thermal insulation properties and can enhance the regulation of temperature and humidity, hence they show strong potential when being used in thermal applications, such as passive houses and low-energy green houses, as less auxiliary comfort will be required [18]. Other benefits of using natural fibres include good adhesion properties, availability, renewability, low-cost, no pollution or toxins are produced [15], biodegradability, easy extraction, and high toughness [14]. Furthermore, plant materials and fibres can retain dust, reduce noise levels and can help act as a shield against the sunlight and weathering effects when placed in the structural envelope [18]. Plant fibres in composites can also help lighten the normal dead load of a material as they are lightweight [15]. Cellulose, lignin, and lignocellulose are the terms used for the fibres that are extracted from natural plants [14].

Various natural fibres and their properties are discussed below.

#### 2.1.1. Bast Fibres

Fibres including flax [4], jute [4], hemp [2], diss [2], and kenaf [2] can be categorised and defined as bast fibres. These fibres are extracted from the exterior of various plants [2]. A common mechanical property possessed by these bast fibres is their high tensile strength; in addition, they possess the ability to be a good thermal insulator [2,18].

Jute fibres are vegetable fibres and are one of the most studied natural fibres [19]. Jute fibres are extracted from the exterior of jute plants; the fibres are contained in the bark [4]. Similar to other materials, there are various types of jute fibres and the properties for each type differ [4]. In construction materials, they help form a porous material that is used for filtration, drainage, and soil stabilisation [4]. These fibres have been used in pavement engineering [4].

Flax fibres have been used in composite construction materials to improve ductility and as reinforcement for building materials to improve stabilisation [4]. Kenaf is a cellulose fibre that is readily available in tropical and subtropical climates such as Malaysia [20].

Korjernic et al. researched the development of insulation materials based on natural fibres, particularly for plant façade applications [18]. Samples were made from a thermal binding technique, where the fibres of hemp, flax, and jute were utilised with the waste material called shive. A bicomponent polyester fibre was used to bind all of the elements together. The results showed that the two samples with the best results were the samples composed of 48% and 49% technical hemp fibres. The material that was developed had comparable thermal insulation properties to market and other natural fibre insulation products. The dynamic stiffness measured also showed potential for applications such as floorboards. It can be concluded that if designed correctly, the developed insulation material can be utilised as external cladding for structures. Improvements in terms of the interior hydrothermal conditions, thermal comfort, and reduced humidity can be achieved using this material [18].

#### 2.1.2. Palm Tree Fibres

Palm tree fibres include coconut (coir) fibres, oil palm fibre, and date palm fibre [2]. Palm fibres are inexpensive, abundant, durable, lightweight, have high water absorption, and have relative strength against deterioration [4]. Palm fibres typically have low tensile strength and a low modulus of elasticity [4].

Coir fibres are extracted from the mesocarp section of the coconut [5]. Coir fibres can be separated into two groups: Short fibres if the length is less than 130 mm and long fibres if the length exceeds 130 mm [5]. These fibres are waste products of agricultural purposes that are constantly being produced all around the world [16]. The waste from coconut is usually burnt or dumped, but can be utilised in a productive manner [16]. Coir fibres are strong, tough, durable, abundant, cheap, have resistance against rot and fungi, are difficult to burn [16], and have high elongation [4]. They also have great insulation properties [16], and, when wet, coir fibres maintain most of their tensile capacity [4]. Numerous studies have researched the physical and mechanical properties and behaviour of coconut-reinforced soil [5]. Ghavami et al. discovered that adding 4% of coir fibres to soil blocks prevented all observable cracks and increased the ductility of the construction product [5]. As coir is composed of a high content of lignin, it has a longer service life compared to other natural fibres. This is due to the slower degradation process [2,4]. As the fibres can be effectively used for four to ten years, they can be purposed as temporary reinforcement [4]. It has been confirmed that coir can assist in increasing the compressive strength, water absorption, and the durability and tensile strength of composite materials [4,5]. These values can also decrease depending on the volume content of coir used [4,5].

Date palm fibres are hydrophilic [15] lightweight fibres that possess mechanical properties, such as durability and tensile strength, and are low-cost, easy to procure, and withstand well against deterioration [4]. These fibres can improve the compressive strength and general strength of composite materials as they lock and unite individual particles and collections of particles together [4]. As mentioned previously, the condition of the environment and the time of harvest greatly influence the mechanical and physical properties of natural fibres [5]. When date palm fibres are collected from a decomposed parent tree, the fibre can be brittle, have reduced tensile capacity, lowered modulus of elasticity, and increased water absorption [4].

Boukhatten et al. studied the water absorption and effect of humidity on the thermal conduction and density of a board produced from a mesh made from date palm fibres and mortar [15]. The results demonstrated many benefits from using date palm fibre mesh in the mortar. The date palm fibre increased the total porosity, reduced the density and thermal conductivity, and, overall, created a lighter material [15].

#### 2.1.3. Cereal Straw

Straw is a common material. The group ‘cereal straw’ refers to wheat straw, barley straw, oat straw, and straw [2]. All around the world, straw is grown and harvested as part of agriculture [2,4]. As previously mentioned, straw has a long history, as it was used as reinforcement to strengthen mud bricks during Egyptian times [4]. Bouhicha et al. demonstrated that if an optimized quantity of straw is used, positive effects will be adopted by the reinforced construction material [4]. Cereal straw has shown the potential to decrease shrinkage, lower the time taken for curing, improve ductility, and enhance the tensile and compressive strength [4]. Straw has high water absorption properties [17].

Bouasker et al. investigated the potential of barley and wheat straw for the construction industry [17]. Some possible benefits stated were its energy efficiency and improved resistance against fire. In this investigation, SEM technology was used to analyse ground samples of the straw. The results showed that straw has low bulk density, which can help make lightweight materials and can significantly improve the hygrothermal quality of a structure [17].

#### 2.1.4. Leaf Fibres

The group ‘leaf fibres’ refers to sisal fibres, banana fibres, and pineapple fibres, and all three fibres are extracted from tropical plants [2]. Sisal fibres are a vegetable fibre, and, similar to jute fibres, are one of the most studied natural fibres [19]. Over 57 species of sisal exist, where the fibres are extracted from the leaf component of the plant [5]. The width of the leaf can range from 6 to 10 cm and the length can range from 50 to 250 cm. The environmental conditions, soil conditions, time of harvest, and type of sisal will influence the size of the leaves [5].

Sisal fibres can be classified into three categories based on their length. If the length of the fibre is less than 600 mm, it is a short fibre [5]. If the length of the fibre is larger than 600 mm, but is less than 700 mm, it is a medium sized fibre [5]. Finally, if the length of the fibre exceeds 700 mm, it is a long fibre [5]. The diameter of the fibre ranges from 0.06 mm to 0.4 mm [4]. Sisal fibres have high water absorption properties of about 60% to 70% and they were traditionally used to reinforce plaster sheets made from gypsum [4].

Together with the coir fibres, Ghavami et al. discovered that adding 4% of sisal fibres to soil blocks removed all observable cracks, increased the ductility of the construction product, and, to some extent, improved the compressive strength [5]. Prabakar and Siridihar identified that sisal fibres lower the dry density of soil [4]. Furthermore, up to a certain limit, the shear stress and shear strength of the soil increased concurrently with an increase in the fibre content and length. However, these properties start to decrease once the limit was exceeded.

### 2.2. Synthetic Fibres

Synthetic fibres are manufactured and produced for a purpose. These include steel fibres [4,8,9,10,12], glass fibres [4,9], plastic fibres, both macro plastic fibres and micro plastic fibres [9], and carbon fibres [3]. Different production methods, and base material composition will lead to different mechanical properties of the fibres [9].

Synthetic fibres can be categorised into two types based on their geometry, these are micro synthetic fibres and macro synthetic fibres [9]. The fibres are categorised based on Table 1.

Synthetic fibres are commonly used as reinforcement to reduce plastic shrinkage and control cracks, replacing the traditional wired mesh and steel bar reinforcement [9,12]. By substituting the traditional reinforcement with synthetic fibres the labour, maintenance, and build cost are reduced, and time is saved from the construction works [9,12]. With the continuous enhancement of technology, and as further knowledge about synthetic fibres is studied, the types of synthetic fibres and their properties have continually improved [21]. Various synthetic fibres and their properties are discussed below.

#### 2.2.1. Polymeric Fibres

There are different types of polymeric fibres, including polypropylene, nylon, and polyethylene, which are the most used polymeric fibres for applications [10]. Polymeric fibres can either be micro plastic fibres or macro plastic fibres. There are three main types of macro synthetic fibres: Polypropylene fibres (PP), high-density polyethylene fibres (HDPE), and polyethylene terephthalate fibres (PET) [9]. The type of fibre, and the mechanical and physical properties it possesses, is related to the technique used to manufacture the material [9], as well as the geometry and aspect ratio of the raw materials [10], and even the properties of the recycled material if the fibre was made from a recycled product. They can be used in similar applications to steel fibres [10], but they have the benefit of being lighter in weight and resistant against corrosion [8,9].

Some methods of manufacturing include melt spinning techniques, the film sheet technique, and manual cutting [9]. The melt spinning technique can form polypropylene fibres and polyethylene fibres with a tensile capacity of over 450 MPa [9]. The film sheet technique can form polypropylene, high-density polyethylene, and polyethylene terephthalate fibres [9]. Recycled PET fibres have been made with this technique with a tensile capacity of 420 MPa and a 10 GPa modulus of elasticity [9]. The manual cutting method is the cheapest and can be used to form recycled PET fibres; however, poorer properties and fibres are produced by this method [9]. Recycled PET fibres with a tensile capacity of 160 MPa and a 3 GPa modulus of elasticity have been produced by means of the manual cutting technique [9].

Macro plastic fibres have harnessed immense interest due to their potential sustainability when compared to the more energy consuming steel reinforcement [9]. Polypropylene fibres are among the most commonly used and appreciated fibres and have advantages, such as being noncorrosive [4] and resistant to alkalis [8,9,11], chemicals [4], and chlorides [4], making them a suitable reinforcement fibre for a diverse range of applications. Little volume is required [11], which makes them cost efficient. Polypropylene fibres have great mechanical properties including high tensile strength and high modulus of elasticity [9]. A disadvantage of polypropylene fibres is that they have a relatively low density, which causes floating issues in some composite matrices [9]. They also possess low hydrophilic characteristics [9] as they are hydrophobic [4], which could affect their ability to bond with certain matrices [9].

When compared to polypropylene fibres, high-density polyethylene fibres have a marginally higher density. They also have a higher hydrophilic tolerance [9]. However, limitations for applications arise, as polyethylene has low tensile strength.

Polyethylene terephthalate (PET) fibres have the highest density of the three. They have good mechanical properties, such as tensile strength and elastic modulus, and can mix easier in matrices such as concrete. A disadvantage of PET fibres is that they take a lot more energy to produce due to the production method; hence, these fibres are more costly. Another disadvantage of PET fibres is that they show degradation in alkali environments [9,11], which limits their range of applications. It should be noted that PET fibres can be extracted from recycled bottles, which shows some sustainability potential [11]. Recycled bottles are made from PET strings, which can be used to make PET fibres [11].

Nylon fibres are micro plastic fibres that can improve the characteristics of the composite, such as the tensile strength, toughness, control shrinkage, and more [9]. However, micro fibres do not have as much effect as macro plastic fibres [9].

#### 2.2.2. Steel Fibres

Similar to traditional steel reinforcement, the key characteristic of steel fibres is their high tensile capacity [9]. Steel fibres have been broadly studied in concrete applications [22], hence, they are commonly used to improve the mechanical properties of concrete [10,22].

Research has shown that when steel fibres are used to reinforce concrete structures, there are many improvements in the overall properties [8]. Steel fibres help improve the concrete behaviour in terms of cracking, shrinkage [9], ductility, toughness, resistance to fatigue, and impact and blast loading [8]. Furthermore, strength properties, such as tensile strength, compressive strength, and flexural strength, are increased for the parent material [6,8,9,10,22]. This strength increase is due to the steel fibres’ characteristics of absorbing energy and controlling cracks. Steel fibres can be an ideal additive to specific applications as they possess good electric, magnetic, and heat conductivity [9].

Although labelled with the general title of steel fibres, there are different types with different properties, and hence, they are not all the same [12]. Hooked end and crimped fibres are different types of steel fibres [6]. Physical properties, such as the length of fibre and fibre count, should also be considered upon preparation for use [12]. Furthermore, the effect of adding steel fibres on strength of the cast and printed concrete has been studied. The research has shown the fibres cause an increase in flexural strength and eliminate the strength difference between cast and printed concrete without fibres [23].

Steel fibres can reduce the cost of construction as they can be used to substitute traditional reinforcement, such as steel mesh and steel rebars, which are much heavier, and require more energy, resources, and time to produce [12]. Funds are also saved due to the lower maintenance and labour required, and, furthermore, the safety conditions of the construction site will be improved [12]. The disadvantage of steel fibres is comparable to steel reinforcement, in that it is quite vulnerable to corrosion [9], which may lead to degradation and deterioration of the parent material [9].

#### 2.2.3. Glass Fibres

Different types of glass exist, with various colours, chemical compositions, and characteristics [24]. Glass fibres have great mechanical properties and excel in terms of strength [3,9,25,26], thermal properties [3,25], durability [3], and have good interfacial bonding to the matrix [3]. Glass fibres are most frequently used as reinforcement in resins and composites [3] as they have amazing properties in strengthening composites [9].

Glass fibres are generally used to reinforce polypropylene systems [26]. A composite is formed between the elements to form an excellent material. The resulting composite is cost effective, easy to procure, and possesses the strength and toughness characteristics of glass fibre [26].

For polymeric materials, glass fibres can be added and purposed as reinforcement. The resulting glass fibre-reinforced polymeric material will have improved properties, such as strength and stiffness, and the composite will also perform better in high temperature environments [9,25].

For composite materials reinforced with glass fibre, the mechanical properties of the composite strongly rely on the behaviour and interaction of the fibre with the parent matrix interface [25]. The finest composite properties are typically the result of a strong interface between the fibre and the matrix [25]. Coupling agents that assist in creating a strong bonded interface have been studied, and silane is a coupling agent that is commonly used for this reason [25]. It helps bond and unite the glass fibres to the matrix [25], hence creating a composite with the best properties. Furthermore, glass fibre-reinforced fly ash-based geopolymer concrete has been developed for large scale 3D printing applications with improved mechanical properties upon fibre addition of up to 1% [27].

Glass fibres are produced by means of the spinneret method, which is similar to the production of continuous basalt fibres [3,28]. Usually, overhead gas burners are used to heat the melt during the production of glass fibres. The melting point of glass is around 1400–1600 °C [28]. In comparison to basalt fibres, glass fibres are less resistant to alkali but perform better than basalt when faced with acids [28].

E-glass is a common type of glass fibre. E-glass is composed of four fundamental compounds: SiO_2_ (silicon dioxide), Al_2_O_3_ (aluminium oxide), CaO (calcium oxide), and B_2_O_3_ (boric oxide) [28]. Through previous studies, it was deemed that the Al_2_O_3_ (aluminium oxide) content greatly influenced the mechanical properties and behaviour of the manufactured glass fibre [28]. Due to this known knowledge, there have been experiments increasing the total Al_2_O_3_ (aluminium oxide) content.

Glass fibre composites have excellent properties when considered and designed carefully [19]. Although there are many advantages and benefits of using glass fibres, there are also disadvantages as the production of glass fibres leads to concerns for the environment [3] and questionable sustainability. Manufacturing requires quantities of B_2_O_3_ (boric acid), which may not be sustainable as the compound is limited [3]. The problem of the disposal of glass products and glass fibre composites during the end of life phase is also well known [3,19]. For most glass fibre composites, recycling could be a possibility, however most products are simply burnt or buried [19], which is not good for the environment. Another disadvantage of glass, is that it degrades and has poor resistance in alkali environments as it has poor alkali resistance [9]. This limits them from concrete applications, as concrete has an alkaline environment.

#### 2.2.4. Carbon Fibres

Carbon fibres have been added in materials to form composites with improved properties [3]. The addition of carbon fibres creates a composite that has outstanding mechanical properties [3], performs well in high temperature environments [3,25], and possesses the benefit of durability [3]. Although carbon fibres are quite brittle [29], with careful consideration in the design stage, carbon fibre-reinforced composites have excellent properties [3]. The disadvantages of carbon fibres are that due to their excellent properties the expense of manufacturing carbon fibres is high [3,29], and the bonding between the fibres and material matrix may be difficult to achieve [3].

Similar to glass fibres, although there are many positives and benefits to carbon fibres, the production of carbon fibres leads to concerns for the environment [3] and questionable sustainability. The problem of the disposal of carbon fibre composites at the end of life phase is also well known [3,19]. For most carbon fibre composites, recycling could be a possibility, however most products are simply burnt or buried [19], which is not good for the environment.

### 2.3. Basalt Fibres

Basalt is a dark, fine-grained, volcanic and igneous mineral that is naturally found and readily available on a global scale [3,28]. It is a solidified rock formed from the hot lava of volcanoes [3,28]. Recently, basalt has been widely discussed due to its strong potential as a suitable fibre reinforcement for composite materials [3,28]. Throughout various fields of study, materials that are sustainable have been heavily studied [30]. This includes research on the natural mineral of basalt, due to its attractive and strong properties and cost-effectiveness [29,30].

Basalt has quite a long history. Since Roman times, basalt rock has been used in its natural state for pavement surfaces and as stones used in building [3]. Scientists in the United States studied the rock in the early 1900s, when significant research was done [29]. During the mid 1900s, the United States and the Ukraine Soviet Union heavily investigated the properties of basalt fibres, and, specifically, their potential as a composite material in defence and military applications [3,30]. Having been widely investigated in the civil engineering industry, interest has spread to a diverse range of engineering fields [19]. With a long history, the research of basalt fibres is still a trend today [29].

Composed of a range of oxides and alumina, basalt is a natural mineral rich in chemicals [17]. The main compounds of basalt in order of volume are SiO_2_ (silicon dioxide), Al_2_O_3_ (aluminium oxide), Fe_2_O_3_ (ferric oxide), FeO (ferrous oxide), CaO (calcium oxide), and, lastly, MgO (magnesium oxide) [3,28,30]. The chemical composition of the basalt rock influences the chemical, thermal, and mechanical properties it possesses including the strength [28], density, physical appearance, heat conduction, and thermal stability [3,30]. Based on the chemical composition, basalt is classified according to Table 2.

Basalt fibres can be divided into two categories: Short basalt fibre and continuous basalt fibre [3]. These two types of basalt fibres are produced through different methods and possess different degrees of properties. The main differences between the fibres can be observed in Table 3.

Basalt fibres show a lot of potential as their chemical, physical, and mechanical properties are very good, which make them competitive contenders as reinforcement for composites [3,19,29]. Some of their strong properties are demonstrated below:

- High tensile strength [3] and modulus of elasticity [29,30,31].
- High resistance to weather and acidic environments, and some alkali resistance [3,29].
- Good thermal properties and stability, and can tolerate and perform well in high temperature environments [3,19,29,30,31].
- Good electric, electromagnetic, and sound insulation properties [3].
- Good resistance and stability against corrosion, chemical attack, impact load, and fire [3,19,29,30,31].
- Good adhesion and abrasion properties with the ability to mix well with matrix materials [3,30].
- Nonreactive [29] and noncombustible [30].
- Low absorption of moisture/water [3] and thermal conductivity [29].
- Absorb sound and vibration isolation [3].
- Resistant to radiation and UV light [3].
- Strong, hard, and rigid [3].
- Improved strain failure [3,29].

Basalt fibres are also:

- Easy to produce and process [29].
- Cost-efficient/inexpensive [3,19,29].
- Used to form lightweight composites with excellent properties [3,29].
- Can be recycled for reuse [3].
- Require no chemicals or additives [19,31].
- Natural [19].
- Biodegradable [29].
- Ecologically clean, easy to handle, and non-toxic [3,29,31].
- Can be used in a diverse range of applications [29].
- Titled as green [29].

As mentioned in Table 3, short fibres and continuous fibres are made by means of different methods and possess different properties. Short fibres are produced using the melt blowing technique and continuous fibres are formed using the spinneret method [28,30]. The basalt rock must be classified as acidic basalt with over 46% of silicon dioxide to be suitable to form continuous fibres [28].

With crushed basalt rock, short basalt fibres can be made through a single step using the melt blowing technique [28]. In this technique, the crushed basalt is melted and poured on top of a set of centrifugal cylinders that are made from steel [28]. Through the air pressure made by air jets, the melt is blown off from the cylinders and solidifies into fibres [28]. Through the spinneret method, better quality continuous fibres are produced [28], but this method requires more time and energy. Broken basalt stone is melted in a rhodium-platinum pot and the melt is then streamed towards a spinneret that is made from an equal material [28]. The spinneret process will then begin, which will result in a spool of continuous basalt fibre [8]. It can be noted that nothing is added to the molten basalt in either method of production. Through the different methods, short and continuous fibres with different properties can be produced.

Sustainable and environmentally friendly materials are key factors to consider in every industry. Basalt fibre is completely natural and has been labelled as a green industrial material, and is informally known as the “twenty-first century nonpolluting green material” [3]. It has achieved this title for many reasons. Basalt fibre can be termed as sustainable, as the raw material is readily available and during the manufacturing procedure absolutely nothing is added to the molten basalt [3]. This means that basalt fibres are completely natural [31].

Basalt fibres show significant environmental benefits, as the recycling at end of life is much more efficient than that for other fibres [3]. The melting point of basalt is very high, hence when a composite containing basalt fibre is recycled, the basalt in the form of powder is left over from the process and obtained. This powder still has great value and can be reused in applications [3]. Basalt products are safe [30]. They are nontoxic, noncancerous, do not react with air or water, are noncombustible, fire proof, and do not have harmful reactions with chemicals [3,30].

Basalt fibres have been used in a broad range of applications, and studies continue to improve their properties and their applications around the world.

Notwithstanding the many benefits when using basalt fibre, there is a disadvantage. It has been found that independently basalt fibres do not perform well in alkali environments [7]. They do not have great alkaline resistance. Nevertheless, basalt fibres have been successfully used in alkali environments before, although alteration or certain additives may be required.

### 2.4. Waste Materials and Fibres

The increase in waste material volume with limited landfill space is a well-known issue, that continues to escalate due to the growth in the population. By utilising waste materials and fibres, the volume of waste that ends up in the landfill can be greatly reduced, and the energy used for the combustion process of landfill materials can also be saved. In this section the types and potential of waste materials and fibres are discussed.

Waste materials contain fibres that could potentially be utilised in applications as a cost effective and sustainable option [32]. Waste materials are classified as secondary materials, as they are recycled, nevertheless, they show potential in replacing primary virgin fibres for specific applications [33]. This can save raw energy, resources, and the extraction processes that are required when procuring primary materials and fibres [33]. There will also be a reduction in pollution and emissions.

Tyres and carpets are objects that can be utilised for their fibres [32]. For tyres, the rubber and steel components can be repurposed and recycled, which leaves the polyester fibre remaining [32]. The rubber component of the tyre is lightweight, has low thermal conductivity, high hydraulic conductivity, and has a high shear strength when the strain is large [33]. Waste carpet can be cut into smaller components and used as polyester fibres [32]. These fibres can help improve the overall performance of the composite material by enhancing properties such as the stability and toughness [32].

Natural hair can be classified as both a natural and a waste product. The fibre of hair can be used in composites or as reinforcement to improve properties such as strength, stability, and bearing capacity, as well as reduce the formation of cracks [34]. Other natural fibres including coconut fibres are also a waste material.

Glass is a material that can be continually recycled and reused while maintaining high quality characteristics and properties [33]. However, there are limitations towards the quantity that can be recycled. For example, in the United Kingdom, only about a third of waste glass materials are recycled due to limitations on the infrastructure used for the collection process [33]. If a more efficient collection process is developed around the world, more waste glass can be recycled. The recycling of waste glass materials for glass making applications conserves the raw materials, energy, and resources that are normally used for primary glass [33]. There are many types of glass with various colours, chemical compositions, and characteristics [24], and the colour of the waste glass influences whether it is appropriate for recycling and re-use. Glass that cannot be recycled due to its colour can seek other industries, such as the construction industry [24].

Steel slag is a by-product from the industrial production of steel [33]. Unlike waste glass and other waste materials, steel slag can be easily collected from the controlled environment of a steel manufacturing plant [33]. Steel slag has good mechanical properties, is rough in surface texture, hard, and has an angular shape. The specific gravity of steel slag is 3.2–3.6 g/cm^3^ [33].

Research and investigation of recycled plastic fibres has been undertaken, however, there is a risk if the prior history of the plastic is not known. If it was procured from an uncontrolled environment, the resulting properties of the recycled fibre may be unstable and inconsistent [9]. Recycled plastics have been used to make products such as furniture, insulation products, ducts, pipes, and more [33]. Waste recycled plastic bottles can also be utilised for their polyethylene terephthalate fibres [11].

Another waste material that is abundantly found but not easily disposed of is cigarette butts. Cigarette butts show potential in causing severe damage to the environment, as cigarette smoke contains over 60 cancerous chemicals, where some may be entrapped in the butts [35]. These entrapped chemicals could possibly contaminate the surrounding area, as the chemicals may leech out. This makes the cigarette butts difficult to recycle. The filter of a cigarette is composed of cellulose acetate. Cigarette fibres can be considered to be cellulose fibres, as they have been made from highly purified cellulose fibres that are acquired from wood pulp, by acetylation [35]. The disposal of cigarette butts in landfills and the incineration of the waste product are not sustainable, economically efficient, or safe [36]. For these reasons, studies on the incorporation and utilisation of cigarette butts recycled in certain applications have been studied by various researchers.

## 3. Applications of Fibres in Construction Material

In a diverse range of industries including the construction industry, the applications of fibre-reinforced materials within composites has consistently broadened and expanded all around the world [3]. When comparing the properties of plain or ordinary materials to fibre-reinforced materials, a wide range of benefits exists. Such positives include:

- Improved characteristics and properties, such as strength, toughness, durability, rigidity, and ductility [3,29].
- Improved resistance and performance in different environments, and against physical and chemical corrosion and other attacks [3,29].
- Improved stability.
- Improved thermal properties and operating temperature [3].
- Reduction of heat conductivity [3].
- Reduction of the specific weight and density resulting in a lightweight product [3] that is both energy and cost-efficient.
- Reduction and lower cost of design and installation [3], as fibres can replace traditional reinforcement methods.
- Reduction of the volume of landfill and saving of energy if a waste product is utilised.
- Prevents the occurrence of shrinkage, cracks, spalling, and swelling.
- Improved environmental-friendliness, economic efficiency, and sustainability [3], particularly if natural, energy efficient, or waste fibre is used.

Different fibres can be used for different purposes and for different applications [29]. Fibres have also been used to develop high performing composites with other matrices [26], which can be used for various applications. The following section discusses the applications of fibres in a handful of construction materials, such as normal concrete, asphalt concrete pavements and binders, soil, earth materials, blocks and bricks, composites, and other industries.

### 3.1. Fibres in Normal Concrete

One of the main construction materials used in building and infrastructure applications all around the world is concrete. Concrete can be used to form many shapes, is resistant to fire, has high durability, and is easy to make [8]. Concrete is strong in compression and weak in tension [8,22], hence, for better performance it requires reinforcement to counteract its weakness. For a basic mixture, the dry ingredients consist of aggregates and cement, and water is used to combine these components together [9]. Portland cement is commonly used in concrete mixtures.

Concrete can be used to form nonstructural and structural elements that support different load capacities [8]. Concrete is used in pipeline infrastructure, road infrastructure, transport infrastructure, and buildings. It is commonly used for slabs-on-ground, industrial slabs, pavements, floors, overlays, columns, shotcrete, tunnel linings, and much more [6,8,10]. In concrete, fibres can be used in their current state, or used to form shapes, such as reinforcement bars, to replace the traditional steel bars and mesh [30], which are heavier and require more energy to produce [9].

Through various studies, it is clear that fibres greatly improve the properties of concrete. Over half of fibre applications are for concrete slabs-on-ground [10], as they reinforce the low tension capacity of concrete [9,22]. One of the major reasons for reinforcing concrete with fibres is to control cracking [8,11]. Other benefits of using fibres in concrete include improved resistance towards cavitation, abrasion, and erosion [8], as well as toughness and ductility [6,8]. The slab thickness can also be reduced, which results in a lower quantity of concrete required, and a larger allowable spacing between the joints can also be applied [6]. It should be noted that when fibres are added to the concrete mixture, the fibre content should be carefully decided, as the content influences the workability of the mixture [8,9,11]. To improve the workability, additives may be required.

Steel fibres are largely used in concrete to improve the mechanical properties of the concrete [10], in fact, they are the most used fibres [22]. Properties, such as flexural strength and ultimate load capacity [6,10], are improved, as steel fibres have good absorption of energy and help control cracks [9]. Toughness, abrasion, and resistance to impact are also improved [10]. Steel fibres are also quite unique and can be used for specific applications, as they possess magnetic, heat, and electric properties [9]. Enlargement of the slab size is also possible through the addition of steel fibres. These benefits and improvements in the properties depend on the volume of steel fibres used and the aspect ratio [10]. However, using steel fibres has a disadvantage, which is similar to that of traditional reinforcement, the fibres have corrosion issues [9].

In concrete, synthetic fibres tend to be used to reduce plastic shrinkage and control cracks [9]. Polypropylene are the most used macro plastic fibres [9], and are popularly used in concrete and mortar applications to manage and lower the formation of plastic shrinkage and cracking [10,11]. Concrete reinforced with polypropylene fibres shows a great improvement in certain properties [9] including improved strength, toughness, resistance to impact, and water tightness [10]. Polypropylene fibres also show a lot of potential in concrete applications, as they are resistant to alkali [9,11], have a competitive cost, improve abrasion resistance [21], reduce spalling, and improve the long-term strength of heated concrete. Some downsides to using polypropylene fibres in concrete, are that due to their low density, floating issues within the composite matrix may be evident, and they may reduce the workability and bonding of concrete due to their low hydrophilic characteristics [9].

Nylon fibres are micro plastic fibres that can improve the characteristics of concrete, such as the tensile strength and toughness, as well as control shrinkage and more [9].

It should be noted that the fibre content and the type of synthetic fibre used is very important, as there has been research that presented minimal improvement in properties, such as toughness [10] and strength [11], when synthetic fibres with a low modulus of elasticity were incorporated into a sample of concrete at a volume content of less than 0.5% [10]. However, improvements in terms of cracking properties and impact resistance are still possible with this type of synthetic fibre [11]. Studies have also shown that other synthetic polyester fibres are not durable and degrade in alkaline environments [11], which strongly affects their potential in concrete applications.

Many studies have shown the benefits of using synthetic fibres in concrete applications. Roesler et al. studied the effects of synthetic fibres for slab-on-ground concrete applications [10]. The fibres used were primarily polypropylene and polyethylene. Large-scale load testing was undertaken to compare the behaviour of nonreinforced slabs with that of synthetic fibre-reinforced slabs [10]. A positive outcome from the addition of synthetic macro fibres was demonstrated. For both centric loaded slabs and edge loaded slabs, the ductility, flexural cracking load and ultimate capacities significantly increased for the fibre-reinforced samples [10]. Fibre contents of 0.32% and 0.48% were used, where the higher fibre samples performed better. No effect was seen for the tensile cracking load [10]. The results showed that the increase in the flexural and ultimate capacities was due to the ability of the fibres to efficiently distribute the load across the slab during the formation of the cracks [10]. Simple supported beam tests undertaken by Roesler et al. also demonstrated the ability of the fibres to increase the toughness of the concrete [10].

Alani and Beckett also studied the mechanical properties of synthetic fibres in concrete slabs using Barchip Shogun to compare their performance to that of steel fibres [37]. A quantity of 7 and 40 kg/m^3^ of synthetic and steel fibres were used, respectively, which is quite a significant difference. The experiment showed that the failure value of the punching shear for the synthetic fibre-reinforced slab was similar to that of the steel fibre, and visible cracks did not form [37]. The results demonstrated that macro synthetic fibres performed similarly to the steel fibres [37]. However, it should be noted that the Shogun fibres have a relatively low melting point, which may be an issue for some applications [37].

Behfarnia and Behravan studied the performance of high performance polypropylene (HPP) fibres in concrete lining applications of water tunnels [8]. The results showed that HPP fibres greatly improved the properties of the concrete lining, including the tensile and flexural strength, toughness, and absorption of energy [8]. Another study stated that the use of steel fibres in concrete linings improves certain properties, such as crack control, toughness, ductility, and strength, and improves resistance in terms of abrasion, impact, and fatigue [8]. Furthermore, when comparing the two fibres, HPP fibres are lightweight and corrosion resistant. These studies show that in tunnel linings, fibres can improve durability and minimise permeability and cracking.

In other applications, such as shotcrete, high performance polypropylene (HPP) fibres have been used to improve the properties, such as the ductility, toughness, load capacity, and energy absorption of the material [37].

Modifications can also be made to synthetic fibres to provide further improvement in the concrete applications [21]. Petkova et al. made modifications to polypropylene fibres by adding nano-additives. The goal was to observe the changes in the properties of the fibres with the addition of the additive. The results showed that nano-additives can assist in improving normal polypropylene fibre-reinforced concrete, as the mechanical and thermal properties can be improved due to increased adhesion [21].

Glass fibres also show potential, however they have low resistance to alkali environments [9]. Khan and Ali investigated the use of glass and nylon fibres for controlling early age micro cracking of concrete bridge decks [38]. The results showed that the addition of the fibres, when compared to the control sample, had a reduced compressive strength and prior cracking energy absorption, but an increase was observed in toughness and the flexural and splitting tensile stress [38]. Overall, it was determined that the addition of nylon and glass fibres is appropriate for controlling the early age micro cracking of concrete bridge decks [38].

Pelisser et al. studied the physical and mechanical properties of recycled polyethylene terephthalate (PET) fibres in concrete environments [11]. PET is a fibre that can be extracted from the waste material of recycled bottles [11]. Tests were undertaken after 28 days and 150 days. At 28 days, the PET fibre-reinforced concrete, excluding the lowest fibre content sample, showed an increase in flexural strength, resistance to impact, and toughness [11]. However, after 150 days the results changed, as the PET fibres slowly degraded over time in the alkaline environment of the concrete [11]. No effect was seen on the modulus of elasticity or the compressive strength [11]. Through this study, PET fibres demonstrated their potential for sustainable outcomes, perhaps in different applications. Further study was suggested on PET Fibres.

Similar to the study of Roesles et al., Altoubat et al. studied the effect of steel fibres for concrete pavement applications [6]. Small and large-scale testing was undertaken. Hooked end steel fibres of 0.35% volume content and crimped steel fibres of 0.5% content were added to reinforce concrete slabs [6]. The results showed a great increase in the flexural and ultimate capacity of the concrete slab [6].

Rolling made a summary that showed that the flexural strength of concrete slabs increased by 35–70% when steel fibres of 1–2% of volume were added [6]. Improvements in the ultimate capacity were also evident [6]. Results from using a high content of steel fibre in thin layered concrete showed a decrease in the slab thickness by 30% to 50%, which was derived from Parkers design [6]. Materials are saved when using fibres.

Through Lloyd’s experience of using steel fibres in slabs-on-ground applications, he compared the typical steel reinforcement with that of steel fibres [12]. The results showed that the use of steel fibres reduced cracks and curling, and the joint spacing for the slab could be enlarged [12]. Positive benefits were noticed in safety, reduction of time, and a reduction in maintenance and cost [12]. However, it should be noted that the fibre count and the type of fibre is key in concrete floor slab applications [12].

Basalt fibres show a lot of potential as they have many benefits and are cheap. Minimal research has been undertaken to examine basalt fibres in alkaline environments [7]. Recently, Myadaraboina et al. investigated this topic and stated that basalt fibres may not be suitable in concrete applications due to the alkaline environment [7]. It was confirmed that the basalt fibres did degrade in the alkaline environment of the concrete and they recommended that treatment or modification is applied to the fibre before applications in alkaline environments [7]. With that being said, basalt fibres have been successfully used for various concrete applications, resulting in strong outcomes [30].

Li and Su investigated basalt fibres in geopolymeric concrete [30]. The results showed great improvement in the capability of the concrete concerning deformation and absorption of energy [30]. Dias and Thaumaturfo also studied basalt fibres in geopolymeric concrete [30]. The results showed that the fibres strengthened the concrete and increased the toughness [30].

Jiang et al. studied basalt fibres in cement mortar and confirmed that the incorporation of the fibres minimised dry shrinkage [30]. It should be noted that the compressive strength was higher for the fibre-reinforced sample compared to that of normal mortar prior to 28 days. After this period, the basalt fibre-reinforced mortar had marginally less strength than the nonreinforced mortar [30]. Although not confirmed, the poor resistance of basalt fibres to alkali environments may be the cause of this finding. Jiang et al. investigated the mechanical properties of basalt fibres in concrete, and found that the basalt fibre-reinforced concrete had drastic improvements in terms of toughness, tensile capacity, and flexural strength [30]. Both the volume of fibre and length of the fibre sample affected the properties of the concrete [30]. Furthermore, Kabay discovered that even minimal contents of basalt fibre showed benefits in respect of the mechanical properties of the basalt fibre-reinforced concrete [30].

Polymer bars that were reinforced by basalt fibres were investigated by Zhu et al. The results showed that polymer bars had excellent properties including resistance against corrosion and great durability, enabling it to replace steel bar reinforcement in concrete applications [30]. It can be noted that the ratio of basalt to steel reinforcement is 1:9.6 [3]. This demonstrates the economic efficiency of not just basalt fibres, but fibres in general, as fibres are generally lightweight [3]. There will also be a significant reduction in the carbon dioxide emissions and energy used for the production of traditional reinforcement [3].

Other fibres, such as natural fibres, typically have poor durability, which make them nonideal for the environment of normal concrete. Jiao et al. investigated the use of cellulose nanofibres in cement paste [39], and the results showed that with the incorporation of 0.15% volume, the fibres increased the flexural strength by 15% and the compressive strength by 20%. The increased degree of hydration and densification of the cement structure due to the fibres were the reasons stated for this improvement [39]. However, no information was given about the future durability of the construction material.

Waste materials, such as the crumb rubber of tyres, have demonstrated recyclability when utilised in concrete slabs with high strength. Previous experiments and studies have affirmed that when crumb rubber was recycled in the concrete slab, the slab had improved resistance against fire and the spalling impairments created by the fire were reduced [40].

### 3.2. Fibres in Asphalt Concrete Pavements and Binders

Asphalt concrete is a primary element used for the construction of road infrastructure and pavements all around the world. The design of asphalt pavement is very important as it helps improve the performance, service life, and economic efficiency of the road network [41]. Asphalt concrete is a type of asphalt pavement [41].

Hot mix asphalt (HMA) is a classification of asphalt where a high temperature is maintained to mix, spread, and compact the asphalt [40]. HMA consists of continuous particle size distribution of aggregates and filler, with low contents of air voids [40]. Stone mastic asphalt (SMA) is a common type of mixture [42]. SMA is commonly used as it can improve resistance towards rutting [20]. Rutting is a known issue for asphalt pavements. This is when there is an abnormal deformation within a smooth surface, such as an uneven surfaced road. For same grades of aggregate and gap-graded SMA mixtures [32], fibres have shown many benefits including the reduction of deterioration and demonstrated improvement in terms of the stabilisation of asphalt [42].

The primary purpose of adding fibres to asphalt mixtures is to increase the toughness of the HMA, improve its resistance to fracture cracks, and help stabilise asphalt binders [32]. The use of fibres can improve the down drain characteristics of the mixture and even prevent the occurrence of down drain [32,43]. Down drain is when the bitumen of the mixture separates itself from the binder during the mixing process, which lead to many issues [20]. Some problems of asphalt pavements include permanent deformation, rutting, and cracks formed due to fatigue, thermal complications [42,43], ravelling because of oxidation, and binder hardening [40]. Durability is also an issue due to the heavier demands of modern traffic [41].

Previous investigations have shown that the addition of fibres to pavement applications and asphalt mixtures increases the strength properties, changes its visco-elastic behaviour, and improves the complex modulus, performance in different environments, the coherence of flow, and controls cracks [42]. Vulnerability to moisture, the reduction of creep, toughness, tensile strength, elasticity, and durability are also improved [44]. From these combined improvements, the issue of rutting is reduced [42].

Cellulose and mineral fibres have been commonly used in asphalt concrete pavements with the purpose of stabilisation [32] and enhancing the resistance of the mixture to rutting [43]. The well-known and suggested volume of cellulose fibres within a SMA mix is 0.3% [43].

Synthetic fibres and basalt fibres have shown benefits when added to asphalt mixtures. Freeman et al. found that polyester fibres in asphalt mixtures improved the toughness properties of the asphalt [32]. Serin et el. investigated the use of steel fibres in asphalt mixtures [42]. The surface layer of the pavement was subjected to traffic loads, and steel fibres were incorporated into the binder course to investigate their benefits [42]. The results showed that the incorporation of steel fibres had great benefits in terms of stability [42]. Manoj Kumar T. et al. studied the characteristics of the SMA mixture when basalt fibres were added [43], and then compared them to those of cellulose SMA mixtures. The results showed that there was a significant improvement in the properties of the basalt-added SMA mixture in comparison to the commonly used cellulose SMA mixture [43]. The addition of basalt fibres also increased the SMA mixtures performance against rutting and down drain [43].

Chen and Xu investigated five types of fibres, and their potential as stabilisers and reinforcers for asphalt binders [44]. A range of experiments and SEM was used to obtain the results [44]. Two different polyester fibres, polyacrylonitrile, lignin, and lastly asbestos fibre were tested [44]. It was concluded that for all the fibres, the dynamic modulus, resistance to flow, and rutting was enhanced [44]. The asphalt binder was successfully reinforced by the fibres [44]. The asbestos and lignin fibres showed higher absorption capabilities, whereas the polyester and polyacrylonitrile demonstrated stronger networking and bonding capabilities [44]. However, it was stated that there was a restriction on the tests that could be performed, hence, further research could be undertaken [44].

Natural fibres have shown benefits when added to asphalt mixtures. Arshad et al. studied two kinds of cellulose fibres in a SMA14 mix, using synthetic fibres and kenaf fibres to stop binder drainage [20]. The results demonstrated that the kenaf fibres performed better in retaining the binder of the SMA14 mix, hence, it can be used as a cheaper and more sustainable substitute to synthetic fibre for this specific mix [20].

Qiang et al. studied the performance of straw composite fibres on SMA pavements [41]. Qiang et al. also analysed polyester, polypropylene, and lignocellulose fibres under the same scope [41]. Their results showed that the dynamic stability starting from the highest was polyester, polypropylene, pavement straw composite fibres, and lastly the lignocellulose fibres [41]. The highest and maximum tensile stress and bending strength was recorded from the highest performer, which was polyester, polypropylene, lignocellulose, and then the pavement straw composite fibres [41]. The optimal pavement straw composite fibre content was 0.28% in the mixture of asphalt [41]. It should be noted that the straw fibres did increase the compressive and tensile strength of the pavement, and that the distribution of the straw fibres was not ideal as they were not uniformly distributed [41]. The purpose of the study was to research the potential of pavement straw composite fibres as a sustainable and economically efficient fibre for asphalt pavements [26].

As sustainability and utilising waste materials is becoming a popular field of study, various researchers have investigated the use of waste fibres in asphalt pavements and binders. Putman and Amirkhanian studied the use of waste fibres in SMA mixtures, and compared it to the commonly used cellulose for the same application [32]. Tyre, carpet, and polyester fibres were used [32]. The results showed great potential for the waste fibres, as they increased the toughness, tensile strength, stopped down drain from occurring, and provided stability to the mixture [32]. The optimal asphalt content was also lower, when comparing the waste fibres to the cellulose fibres, which shows great potential for the reduction of cost if less asphalt is required [32]. Overall, the study provides a cost-effective alternative and demonstrates sustainable value [32].

With the constant construction and required maintenance for roadwork, a high quantity of quarried aggregates are required [33]. Recycled waste materials in substitution for newly produced aggregates can save energy and reduce the volume of accumulating waste in the landfill [33]. However, the quality, performance, and costs required have been questioned [33].

Huang et al. reviewed the use of recycled waste materials in asphalt pavements [33]. Waste materials of glass, steel, tyres, and plastics contain fibres and were the focused materials of this review. It was concluded that the steel slag was a great substitute for the coarse aggregate in surface asphalt, as it has excellent mechanical properties including strength and skid resistance; however, there are concerns due to its electric conductivity and concentration of chromium. A high content and particle size were recommended. The crushed waste glass in the asphalt was seen as a safety risk from a technical perspective. Waste tyre materials can be used as either a binder element or an aggregate element of asphalt. It was summarised that tyre rubber as a binder in asphalt mixtures helps improve durability, low temperature performance, and reduces noise and the formation of cracks. When low density polyethylene from recycled plastics replace 15–30% of the aggregate quantity used, improvements in terms of rutting, crack control, and performance may be possible. Nevertheless, these are dependent on factors such as the design and the particle size. Overall, it was concluded that the processing cost may often be higher than the typical cost of virgin aggregates, and there are also concerns over the potential effects, such as run-off pollutants and leaching [33]. This may need to be further studied, although it does show further applications and utilisation of waste materials in asphalt applications.

When glass particles are recycled in asphalt an impervious barrier is formed, which helps reduce the time required for the surface to dry when it rains [40]. Coloured waste glass could be utilised for this application. 

Lastly, Mohajerani et al. integrated encapsulated cigarette butts in asphalt concrete and investigated the physicomechanical properties [40]. Encapsulating the cigarette butts inhibited their interaction with fluids. Two methods were investigated. One method used different classes of bitumen to encapsulate various quantities of cigarette butts (10, 15, and 25 kg/m^3^), where they were added to a Class 170 bitumen modified mix of asphalt concrete. The other method used paraffin wax to encapsulate a set quantity of 10 kg/m^3^ of cigarette butts, in which they were added to C170 and C320 classes of bitumen modified mix asphalt concrete. The results showed that for method one, when 10 and 15 kg/m^3^ of cigarette butts were used in the mixture, the asphalt achieved acceptable properties for light, medium, and heavy traffic conditions. The class, amount of bitumen, and amount of cigarettes encapsulated influenced the properties of the asphalt concrete. For the second method, when 10 kg/m^3^ of cigarette butts were encapsulated with the wax and incorporated into the bitumen asphalt mix, due to the lack of stability, only the requirements for the light traffic conditions were satisfied. By adding encapsulated cigarette butts in the mix, there was a reduction in the bulk density of the asphalt concrete and an increase in the porosity, which decreased the thermal conductivity [40]. Further studies for these methods of encapsulation and the incorporation of encapsulated cigarette butts in asphalt and different types of construction applications were recommended [40].

### 3.3. Fibres in Soil

Typically, soil is weak in tension and shear strength [4,45]. Soft soils in particular can be quite vulnerable to deformation [45]. The purpose of soil reinforcement is to enhance the properties of soil [4]. There are four common varieties of soil: Clay, sand, silt, and gravel [4]. The environmental conditions and the climate of the area influence the properties of the soil [4].

Fibres can be used to improve the properties in which the soil is poor, such as tensile capacity, shear strength, ability to compress, density, and hydraulic conductivity [4]. Shrinkage, which influences the formation of soil cracks, can also be reduced or even stopped [5]. By improving these properties, soil and the surrounding slopes can be stabilised, bearing load capabilities can be enhanced, and there can be a reduction of both settlement and deformation along a lateral plane [4]. A diverse range of natural and synthetic fibres has been used to reinforce soils for different applications [4]. Some applications include pavement layers, retaining walls and rail embankments, slopes and foundations, and earth quake engineering [4].

When a construction project is located near poor soil, it is important to either remove or improve the soil [45]. Removal of the soil is an expensive process when factoring in the cost and time required for the procedure [45]. Hence, improvement of the soil may be a more cost-efficient way to handle poor soil. If neither the removal or improvement of soil are undertaken, after the construction, additional maintenance work and monitoring may be required to reduce possible risks [45].

In the geotechnical industry, expansive soils are a known issue and one of the biggest challenges. These soils have low mechanical properties, are low graded, and inadequate for subgrade applications [46]. In the natural environment, the moisture content will change, and due to this change, the volume of the expansive soil will vary significantly [47]. This sudden change of volume develops an issue called swelling, which becomes more severe as the plastic index increases [47]. Swelling can cause cracks and shrinkage and reduce the shear strength and capacity of the soil [47]. The alternation of the soil is required through a term called soil stabilisation, in which an additive is used to chemically stabilise the soil [46]. Cement, fly ash, and lime are commonly used [48], however concerns arise about the negative impact on the environment and the possible leaching of the chemicals. Other methods to overcome swelling include soil removal or replacement, monitoring and controlling the compaction, thermal assistance, surcharge loading, and the use of supportive reinforcement [47]. More recently, there have been studies using nontraditional stabilisers and fibres to improve the swelling properties of soil [46,47].

Although not a fibre, Soltani et al. recently studied the use of sulphonated oil to stabilise expansive soils [46]. Concentrations of 0.25–2.5% of the total water mass were tested, where 1.25% demonstrated the best results. The use of sulphonate oil enhanced the strength properties and reduced the swelling pressure of the soil, where the swelling properties depended on the concentration of oil used. The curing time did not alter the results. Overall, it was determined that the use of sulphonate oils can be an economical and more environmentally friendly substitute to the typical additives used to stabilise expansive soils [46].

The mechanical properties of soil can be improved and stabilised by using different techniques [45]. Previous studies have shown that the use of natural fibres in poor soils helped reinforce and improve the mechanical behaviour of the soil, even if a small volume of fibre was used [45]. Waste materials and synthetic fibres have also been previously investigated and it was determined that these fibres assisted in enhancing the strength of the soil as they strengthened and developed friction interlocks and bonds between the soil particles [45]. In respect of fibre length, short fibres were reported to be more effective in the soil [4].

Fibres can be used to repair slopes in localised areas [4] and improve the bearing capacity, shear strength, reduce settlement, and stop deformation for the soil foundations [4]. Natural fibres have been used to reinforce soil. When embedded in clay soil, coir fibres can maintain about 80% of their tensile strength after half a year [4]. Jute fibres are used for stabilising soil, and for filtration and drainage applications [4]. Gosavi et al. mixed nylon fibres and jute fibres, and also used coconut fibres to reinforce soil. The results showed that the California bearing ratio (CBR), which evaluates the strength, increased by 50% and 96%, respectively, when compared with the control sample, where the optimal fibre content was 0.75% [4].

Synthetic fibres, specifically polypropylene fibres, have demonstrated positive benefits when used as reinforcement for soil. Geofibre is a term that is widely used in soil reinforcement, and mainly refers to polypropylene fibres [4]. For laboratory experiments, polypropylene fibres are commonly tested to reinforce soil. Tests and experiments have shown that when polypropylene fibres are used to reinforce soils, there are improvements in the strength behaviour and ductility, and a reduction in the shrinkage, degradation, and swelling of expansive clay [4]. Polypropylene fibres have been used to reinforce soil walls, where they have enhanced the stability, reduced the wall displacement, and reduced earth pressures [4]. Polypropylene fibres have also been used as reinforcement to repair roadway slopes and embankments, and they enhance the performance of slopes [49]. Short fibres were reported to be more effective in the soil [4].

Tests and experiments have shown that the addition of polyester (PET) fibres has enhanced the bearing capacity and stability of levees in terms of seepage and reduced settlement depending on the quantity of fibre used [4]. The results from Maheshwari showed that the bearing capacity and safe bearing pressure of a highly compressive clay soil increased concurrently as the fibre content increased, however, this was capped at 0.5%, and if this volume content was exceeded, there would be reduction in the bearing behaviour [4]. Kim et al. utilised fishing nets that were made from polyethylene, to reinforce lightweight soil [4]. The results showed that 0.25% volume showed the most optimal enhancement of the compressive capacity of the lightweight soil [4].

Glass fibres have been used to enhance the peak capacity of silty sand and increase the unconfined compressive strength of cemented sand [4]. In cemented soils, glass fibres have increased the failure deviator stress and marginally decreased the brittleness [4]. Although not as efficient as polypropylene fibres, steel fibres can enhance the strength properties of soil, and they have been used as reinforcement for soil and cement composites [4].

For pavement applications, Grogan and Johnson studied geofibre in clay, modified sand, and silt soils [4]. The results showed that with the addition of geofibre, there was a 90%, and 60% increase in the failure traffic load capacity for clay and modified sand soils, respectively. Geofibre-reinforced silt soils also showed improvement [4]. Similar to asphalt pavement, rutting, deformation, cracking, stabilisation, and many other enhancements can be developed when fibres are used as reinforcement for soil based pavements [4].

In earthquake engineering, it is important to reinforce the foundations and support the soils that surround structures in areas that are prone to earthquakes, such as Japan. Makiuchui and Minegishi stated that synthetic fibres can be utilised in techniques to reinforce soils for earthquake conditions [4].

When fibres are used to reinforce clay soil, it is common that the strength properties across the sample are nonuniform, as there are variations in the content and distribution of fibres and the density throughout [50]. The clustered fibres that form fibre pockets, and the preparation methods used are also factors of inconsistency [50]. Uniformity is important, as the strength properties of soil correlate to the overall structural integrity [50]. Saad et al. investigated the uniformity of density of carpet fibre-reinforced clay soil, prepared by the method of static compaction [50]. The results showed that the uniformity of density, distribution of fibres, and method of preparation significantly influenced the strength and homogenous characteristics of the reinforced soil [50]. It could be observed, that as the layers of soil increased, the simultaneous static compaction of the soil from both ends improved the uniformity of the sample [50]. Using this method, with five thin layers, uniform density and fibre content was achieved, and there was a 45.5% increase in the unconfined compressive strength of the sample [50]. This increase was due to the properties of fibre and also the uniformity achieved. This study can be used to improve the accuracy of experiments and the use of fibres in soil applications.

Waste materials have shown benefits when utilised in soil applications. Throughout various studies, Mirzababaei et al. have studied and demonstrated the benefits for utilising waste carpet fibres in soils and slopes. Recently, Mirzababaei et al. studied the influence of fibre reinforcement on the properties of the shear strength and void ratio of soft clay [45]. A range of drain reverse shear tests with multiple stages were undertaken to analyse the results. The synthetic fibres of polypropylene were used at volumes of 0.25% and 0.5% and of lengths 6, 10, and 19 mm. The results showed that as the volume and length of the fibre increased, the shear strength improved concurrently, however the benefit was capped at the normal effective stress that was applied at the shearing stage [45]. The shear strength of the fibre-reinforced clay was similar to the control sample when the normal effective stress was high, in that the fibre incorporated did not have much effect at this time [45]. The shear strength and compressibility reduced as the shear cycles increased [45]. The void ratio lowered as the fibres helped increase the friction and interlocking mechanics between the particles of soil [45]. A consistent finding was that as the fibre content and length increased, the effective cohesion of the soft increased, respectively, however the effective internal friction angle did not change much [45].

Mirzababaei et al. also recently investigated two kinds of carpet waste fibres to reinforce and improve the shear strength of clay soil [51]. The soil had a plastic index of 17%. Fibres of 1% to 5% volume were tested using consolidated undrained triaxial compression tests. The results showed that the carpet fibre-reinforced clay enhanced the deviator stress of the soil and the effective shear stress ratio [51]. It was concluded that up to 5% of the waste carpet fibre could be utilised to enhance the shear strength behaviour of clay soil. A sustainable and environmentally friendly method to reinforce weak soil was developed [51].

In a similar study, Mirzababei et al. investigated the use of two different types of carpet waste in two different clay soils [52]. The two soils had a plastic index of 17% and 31.5%. The fibre contents of a dry soil volume of 1–5% were tested by means of a systematic procedure. The results demonstrated that if the carpet fibres were prepared with an equivalent dry unit weight, there could be great improvements in the unconfined compression strength (UCS), the strength decrease after peak could be minimised, and ductile instead of brittle behaviour could be achieved upon failure [52]. The improvements achieved when adding the fibres were strongly influenced by the initial dry unit weight and the moisture content of the clay soil [52].

In another study, Mirzababaei et al. investigated the use of waste carpet fibres as reinforcement for slope applications [49]. An extensive laboratory study and a particle image velocimetry technique was used for this analysis. It could be observed from the results, that the addition of fibres to the model slope significantly enhanced the bearing resistance properties of the soil slope. For example, when 5% fibre content was incorporated, the bearing pressure improved by 145% in comparison to the unreinforced soil slope [49].

Swelling is a large issue for expansive soils. Mirzababaei et al. also investigated the swelling properties of compacted cohesive soil when carpet waste fibres were incorporated [47]. Similar to previous studies, two different types of carpet waste fibres in two soils were tested. The two soils had a plastic index of 17% and 31.5%. The fibre contents of a dry soil volume of 1–5% were tested. The initial compaction condition and moisture content significantly influenced the results. The results showed that the samples formed with optimum moisture content, and at the maximum dry unit weight the swelling pressure decreased dramatically and respectively to the increase in fibres [47]. If the moisture content in the soil was unchanged and the dry unit weight decreased or if the moisture content increased and the dry unit weight remained unchanged, the swelling pressure was reduced [47]. This investigation demonstrated a cost-effective way of addressing the swelling issue.

Besides waste fibres, Mirzababaei et al. also investigated the swelling characteristics of three types of expansive soils reinforced with three different polymers [48]. Furan was tested at 3%, 5%, and 10%, and polymethyl methacrylate and polyvinyl acetate were tested at 1%, 3%, and 5%. The results showed that the free swell potential reduced as the furan content increases, capping at 10% [48]. The remaining two polymers also reduced the free swelling potential of the samples, but the results were not as significant. Aggregation and granular matrices were also developed from the addition of the polymers, where the potential of swelling was lowered [48].

Human hair is another waste material that can be utilised for its properties. Butt et al. investigated the effect of human hair when reinforcing clay soil. The test results were impressive. Enhancements were observed in the strength and stability of the clay soil. The optimum fibre content was 2%. It was suggested that the great properties of human hair fibres could possibly be used to reinforce embankments and help stabilise slopes [34].

### 3.4. Fibres in Earth Materials, Blocks, and Bricks

Earth has been used as a construction material for a very long time and it is still commonly used all around the world, especially in low economy countries [2]. Earth materials show many sustainable and economic benefits, such as reduced burden on the environment, as earth materials are readily available, and, furthermore, less carbon emissions are produced and minimal processing activities undertaken [2,53]. Although soil is within the category of earth materials, this section will specifically review the different types of fibres used to reinforce earth material for construction, specifically those purposed for housing.

Some earth material composites that can be enhanced by fibres are adobe blocks, compressed earth blocks, and techniques such as earth plasters [2]. Adobe blocks are man-made sundried blocks [2], mainly composed from water and earth materials. The types of blocks include clay, mud, soil, and more, which are made from different methods, such as the sundried, baked [54], fired method [16], and the traditional compaction method, where the mixture is poured into a mould, and a vibrator is used for compaction, and then set [53].

Different fibres used in different earth materials show various benefits. These can include increased durability [5], compressive strength [5], tensile strength [53], elasticity [53], layer coherence [53], geometric integrity [53], water absorption [5], thermal properties [54], reduced shrinkage [5], reduced formation of cracks [5], and reduced deadweight due to the lighter weight block [16,53]. Utilising natural fibres in earth material or blocks is sustainable, environmentally friendly, and also saves energy and costs, as the volume required at landfills and the burning procedures used to get rid of the waste will be reduced, or no longer necessary [16].

Straw is a common material that is readily available all around the world [4]. Barley straw fibres are typically used to reinforce and make soil composite blocks [4].

Fibres in mud bricks improve the compressive strength, tensile strength, elasticity, and overall coherence of the brick [53]. The fibres also support the shape integrity of the mud brick [53]. Binici et al. investigated the use of fibres to reinforce mud bricks. Three samples were tested. The base materials were cement and pumice and each sample contained one of the fibres: Plastic fibre, fabric made from polystyrene, and straw [53]. Gypsum, as well as lime, were added to each sample to help with stabilisation [53]. The results showed that the fibres helped improve the compressive strength, the highest being the plastic fibre sample, and the shape integrity of the mud brick [53]. It was concluded that the fibre-reinforced mud brick was very efficient, as any shape could be formed, and a higher compressive strength was achieved for a lower deadweight and cost [53].

Ghavami et al. studied the behaviour of soil blocks reinforced with 4% coconut fibres for one block and the same volume of fibre for a sisal-reinforced block [5]. This study focused on the area of Brazil. The additives of two natural bituminous water repellent materials named piche and cipla were used [5]. These water repellents were used as fibres to take in water and bloat, then dry back up. The change in dimensions forms voids, which potentially leads to shrinkage, swelling, and weakens the overall matrix structure [5]. The compressive strength for both samples increased by a small margin, when compared to the plain soil block, whereas the ductility of reinforced blocks greatly increased [5]. Sisal showed more potential, as it absorbed more water than the coconut, resulting in a denser block. The most important improvement gained from the addition of the fibres was that shrinkage was heavily reduced, or even stopped, hence no visible cracks were formed [5].

Mekhermeche et al. investigated the thermal properties of date palm fibre-reinforced clay bricks for hot climates [54]. This investigation focused on Algeria, specifically the Saharan regions. Local materials of clay, sand, and date fibres were used to form the bricks. Mud containing sand and fibres filled the ground and a baking technique was used to form the bricks in the ground [54]. The results demonstrated that when a higher mass of sand and fibre was used, there was a positive enhancement of the thermal properties [54].

Raut and Gomez attempted to develop a thermally efficient and sustainable brick using local waste materials and fibre [55]. This study focused on the area of Malaysia. The raw materials that were used and procured locally consisted of scarp glass powder, palm oil fly ash, oil palm fibres, crusher dust, lime, and water [55]. These were mixed in a concrete mixer, then poured into moulds and compacted by vibrators. After a day, the bricks were removed from their moulds, set for a few days, and then cured for the standard 28 days. The bricks developed showed great potential and a possible substitute to conventional bricks [55]. The results showed low thermal conductivity, which reduces the surrounding temperature, hence, saving energy as less auxiliary heating or cooling would be required for thermal comfort [55]. The density decreased making it lighter in weight, and the compressive strength also decreased, however, it was still within a feasible limit [55]. A high absorption rate for the initial phase was quite worrisome and may need to be studied, but, overall, a brick that shows sustainable potential with good thermal properties was developed [55].

Namboonruang and Yongam-nuai researched the properties of lime soil bricks reinforced with the natural fibre of cellulose [56]. This study focused on the area of Thailand, where all of the materials were procured locally. A cellulose fibre mix made from waste wood material and leaf aggregates, lime, soil, and cement were the base materials used [56]. Shale and slag were also used. The results showed that an increase in cellulose fibre content caused a reduction of most of the properties including the density, compressive capacity, flexural capacity, and thermal conductivity [56]. There was an increase in the water absorption, which was linked to the voids and weakened properties [56]. Although the brick could not be used as a load bearing brick, bricks with up to 55% volume of cellulose fibres could be used to make bricks for non-load bearing applications [56]. The bricks showed great sustainability as local materials were utilised, and hence, they were low-cost and saved energy. The study concluded that this may be an answer to the building of low-cost housing units in Thailand [56].

Kadir et al. studied the possible use of coconut fibres in fired clay bricks. Coconut fibre fired clay bricks and normal fired clay bricks were formed and compared [16]. This study focused on the area of Malaysia. Fibre volume contents of 1%, 3%, and 5% were tested and the 3% fibre content sample showed the best results. The results showed that the properties of the brick decreased with increasing fibre content, however the 1% and 3% fired clay bricks were still within the compliance range [16]. In conclusion, the slightly lower properties, compared to the cost and energy saved, could be a sustainable and environmentally beneficial trade-off [16]. The reduced strength was likely due to the fact that the temperature used to produce the fired clay bricks was higher than the melting temperature of the coconut fibres, hence the properties decreased.

The build-up of waste materials all around the world is a known issue, as landfill space is limited, and the incineration process requires a lot of energy and produces unwanted emissions. Cigarette butts, which contain cellulose fibres, are one of these waste materials. Mohajerani et al. studied cigarette butts and proposed a solution to the world’s cigarette butt issue by recycling the waste material in fired clay bricks [35]. When 1% volume of cigarette butts was added to the fired clay brick mixture, there was only a marginal decrease in the mechanical properties. The cigarette butt fired clay bricks still maintained acceptable properties for load bearing applications [35]. The thermal conductivity of the brick was also reduced, and overall, approximately 9% of firing energy was saved. It was calculated that if 2.5% of the annual production of bricks incorporated 1% volume of cigarette butts, the cigarette butt problem could be solved [35]. This proposal shows how toxic waste materials can be utilised in brick making applications.

Though not an earth material, red mud is an industrial waste that is a product of aluminium procurement [57]. Dash et al. investigated the mechanical properties of a red mud filled hybridized composite [57]. Fibres of glass and jute, red mud, a resin epoxy, and a hardener were used. The results showed that the flexural strength, tensile capacity, and density of the material increased respectively with the number of reinforcement layers [57]. It was concluded that the studied composite material was suitable for low load bearing applications [57]. This study further shows the utilisation and possible applications for waste materials.

### 3.5. Fibres in Composites

Fibres also form composites with other matrices to improve the properties and characteristics of the composite material. For example, a fibre could form a composite with a polymer to form a polymer composite with excellent properties [19,29]. With the continuous increase in the demands and needs around the world, comprehensive studies have been made to try and improve the properties of epoxy resin matrices and polymer composites [58]. The physicochemical interaction between the components of the composite, the degree of adhesion, and the bonding between the fibre and the matrix are extremely important factors that influence the mechanical behaviour of the composite [26,29,59]. Both synthetic and natural fibres have been used to reinforce other matrices. If a natural fibre, basalt fibre, or recyclable fibre is used to reinforce a biodegradable polymer, a composite that is environmentally friendly can be produced [30].

As mentioned previously, glass fibres are often used as reinforcement in resins and composites [3]. A composite is formed between the elements to form an excellent material. For polymeric materials, glass fibres can be added and purposed as reinforcement, resulting in a polymer composite with improved characteristics in areas, such as strength, stiffness, and performance in high temperature environments [9,25].

Glass fibres are generally used to reinforce polypropylene systems [26]. Polypropylene resins show a cost efficient and high performance ratio [58]. Etcheverry and Barbosa studied the properties of a glass fibre-reinforced polypropylene when the adhesion was controlled and improved [26]. The results showed that when in situ polymerised fibres were used to form the composite of glass fibre-reinforced polypropylene, the properties significantly improved [26]—the toughness and strength of the material tripled [26]. This study showed the importance of adhesion between the materials. Glass fibres have also been used to reinforce plastic polymers to form the material of fibreglass.

Basalt fibres bond well with matrix materials [30], and they have great mechanical properties, are fire proof, and require no additives during their production [16]. They are also lightweight, have high load-bearing properties [17], can be used in a wide range of temperatures, and have good resistance to chemical attacks and impact loads [31]. Basalt fibres have been widely studied for reinforcing polypropylene and epoxy resin matrix composites [31]. In applications, basalt fibres have been used to reinforce polymers to create thermoset polymers [30]. They have been used to reinforce polyester, epoxy, and vinyl ester resins, where the composite formed is used for the production of a variety of applications [30]. Short-beam tests have also been undertaken, which have demonstrated the good interfacial adhesion and bonding between the basalt fibres and epoxy matrices [30].

An old study concluded that basalt fibres could be a nontraditional material used to reinforce polypropylene composites [58]. Botev et al. investigated whether untreated short basalt fibres provided suitable reinforcement for polypropylene polyester by observing the change in the viscoelastic behaviour of the composite [58]. It could be observed from the results, that the tensile properties of the fibres deteriorated due to the poor properties of the short basalt fibres and the weak adhesion between the polyester and the fibre. To improve the adhesion a coupling agent of PP-g-MA was used, and the improved interactions were visually observed through SEM technology. With the improved adhesion, there was an improvement in the stress capacity at yield, strength, and stiffness [58]. This study also demonstrated the importance of adhesion, and the improvement in the properties of the composites when the adhesion was improved.

Mingchao et al. studied the mechanical properties and chemical resistance of alkali-proof basalt fibre and the characteristics of this fibre when used to reinforce epoxy composites [31]. Basalt fibres were distilled and then boiled in sodium hydroxide and hydrochloric acid to determine whether the fibre was more resistant to alkali or acid; the results showed that it was more resistant to alkali [31]. Basalt fibres were used to reinforce an F46 epoxy resin matrix composite using the hot-pressing method. The composites were then submerged in eight types of chemical medium to determine the chemical and corrosion properties. On the 15th, 30th, and 90th day, the mechanical properties of the basalt composite were measured using the three-point test. An S-2 glass fibre-reinforced epoxy composite was also formed from the hot-pressing method, and its mechanical properties were tested and compared with that of the basalt fibre-reinforced epoxy composites. The results demonstrated that in alkali mediums the flexural modulus remains constant, whereas the strength decreases, while both the flexural strength and modulus decrease in an acid medium [31]. When comparing the glass- and basalt-reinforced epoxy resin composites, it was determined that the mechanical properties for the glass-reinforced epoxy resin were higher, but the interlaminar shear strength was higher for the basalt fibre-reinforced epoxy resin, which resulted in a better formation of the interface for the basalt composite [31]. Hence, basalt fibres can be a suitable alternative to glass fibres to reinforce composites [31].

Park and Jang studied the use of polyethylene fibres and carbon fibres in an epoxy matrix to produce a hybrid laminated composite material [29]. From the results, it was determined that the excellent mechanical properties of the hybrid composite material were strongly reliant on the position of the reinforcing fibre. When the carbon fibre was placed on the outer layer, the composite had very high flexural strength [29].

Natural fibres used to reinforce polymeric composites have been an increasing trend, as they are not dangerous, are low-cost, renewable, and could potentially replace synthetic fibres for some applications [14]. Although durability and degradation are a concern [14], there have been studies to try and improve the durability of the fibres [14]. Natural fibres, such as cellulose nanofibres, have great properties and a high elastic modulus [39]. They have been used to reinforce and improve the mechanical behaviour of polymers including chitosan and polylactic acid [39].

When natural fibres are used to form composites with thermoset matrices, an improvement in the mechanical properties has been demonstrated because of the chemical bonds formed at the interface and the low viscosity of the resin [59]. This allows a good interaction between the fibre and the resin [59]. Natural fibres that are used to form composites with thermoplastics have demonstrated a more environmentally friendly approach [59], as thermoplastics are more recyclable. However, issues may arise, as there are lower interactions at the interface between the natural fibre and the thermoplastic matrix because, typically, the natural fibres have a hydrophilic nature whereas the thermoplastic matrices are hydrophobic [59].

European car manufacturers have been using natural fibres to form thermoplastic composites and thermoset matrices [60]. Natural fibres, including hemp, kenaf, jute, flax, and sisal, are lightweight, and hence, the deadweight of the product can be lowered, there is higher recyclability potential, and lower carbon dioxide emissions and reliance on oil sources [60]. Challenges consist of fibre homogenization, knowledge of combining fibres and crystallization, adhesion and surface interface required between the natural fibre and the matrix, the methods to control moisture, and flame retardant properties [60].

Fuentes et al. studied bamboo fibre-reinforced thermoplastic composites [59]. The thermoplastic polymer films tested were polypropylene, polyethylene terephthalate, maleic anhydride grafted polypropylene, and, lastly, polyvinylidene fluoride [59]. The effect of the physical adhesion on the mechanical behaviour of the composite was analysed. The results showed that if the surface energy of the thermoplastic matrix and the bamboo fibres matched, the composite would have higher adhesion [59]. The surface energy of the bamboo fibres and the polyvinylidene fluoride composite matched and had good wetting parameter combinations, hence there was improved adhesion, which resulted in the best results and improved mechanical properties, such as interfacial and longitudinal strength [59].

### 3.6. Fibres in Other Applications

Outside of the construction industry, many other industries have utilised different fibres for various applications. Such industries include the automotive industry, the power industry, the chemical industry, the petrochemical industry, wind mill blade industry, sports industry, ballistic industry, defence industry, and the aeronautic industry [3,29,30]. A few applications are listed below.

Carbon fibre composites have excellent mechanical properties, hence, they have been heavily used in the automotive industry [29]. When carbon fibres are used as reinforcement, a lightweight material can be formed, however this is typically expensive. For example the deadweight of a vehicle body may be minimised by 40% to 60% [29]. In the aircraft, space technology, defence, and sporting industries, where specific and highly developed applications or technology are required, carbon fibres have also been used [3,29].

Glass fibre-reinforced polypropylene composites have been increasingly used in the automotive industry, as they have excellent mechanical properties, are simple to fabricate, lightweight, and are quite economically affordable [26]. Products, such as bus bumpers and car seats, have been made using these composites [26].

Basalt fibres are used in the automotive industry to make products such as brake disc pads and car headliners. They are especially popular due to their recyclability; high strength; ductility; and resistance to corrosion, impact, and wear [3]. Basalt fibres have been used in the power industry as they have great thermal conductivity and are resistant to corrosion [3], and in the chemical industry to coat and preserve items as they are resistant against wear and chemical damage. In the petrochemical industry, basalt fibres have been used to form pipes as they have low thermal conductivity [3]. In the wind mill blade industry, basalt fibres can be used to make longer blades, thereby increasing the overall potential energy harvested. In the sports industry [3], the fibres have been used to produce equipment, such as skis, bicycles, and tennis rackets [3]. Lastly, basalt fibres have been recently used in defence applications to help reduce the deadweight of heavy vehicles [30].

There has been a growing interest in using natural fibres in various industries including the aerospace and automotive industry [17]. This is mainly due to their cheap cost, being a renewable resource, and their potential in terms of sustainable development [17]. In some applications, they can be a substitute for synthetic fibres [17].

## 4. Discussion

The factors that influence the properties of the fibres, the advantages, disadvantages, and limitations of the different fibres are summarised in this section. Furthermore, important factors to consider when using fibres in construction materials and composite materials are discussed. Although most of this information was discussed earlier, it will be highlighted in this section.

### 4.1. Natural Fibres

With natural fibres, the condition of the surrounding environment at the time of harvest will influence their physical and mechanical properties [5]. This includes the condition of the soil, the process used for the extraction, any treatment undertaken, air humidity, temperature, and more [5].

Natural fibres have many advantages. They are readily available and are easily extracted, abundant, require little to no energy to produce, can be extracted from waste material, are economical and low-cost, and can reduce the environmental impact when used in the construction industry [2,4]. Furthermore, they have good thermal insulation properties [18], adhesion, renewability, no toxins or pollution are produced [15], and they have high toughness, are biodegradable [14], and lightweight [15].

The use of natural fibres to reinforce polymeric composites has been an increasing trend and could potentially replace synthetic fibres for some applications [14,17], thereby promoting sustainable materials and development.

The main limitations and disadvantages of natural fibres is that they typically have poor durability and may naturally degrade over time [9,14]. Another disadvantage of plant fibres, is that they are hydrophilic [15]. There have been studies to try and improve the durability of the fibres [14], which include chemical treatment, coating, and substituting or blocking the hydroxyl group of the organic natural fibres, but further research is required to determine whether the improvement in durability is long-term or just temporary [14]. It has been stated that these methods are quite costly and could be hazardous as leeching, and exposure to biological, chemical, thermal, and UV-related degradation have not be investigated in detail [14]. As natural fibres tend to have a shorter lifespan, they may be limited and purposed as temporary reinforcement [4].

### 4.2. Synthetic Fibres

For synthetic fibres, different production methods and base material composition will lead to different mechanical properties of the fibres [9]. With the continuous enhancement of technology, and as further knowledge about synthetic fibres is studied, the types of synthetic fibres and their properties have continually improved [21].

One advantage of synthetic fibres is that they can reduce the cost of construction as they can be used to substitute traditional reinforcement such as steel mesh and steel rebars, which are much heavier and require more energy, resources, and time to produce [12].

Nevertheless, among the disadvantages of synthetic fibres are that they are man-made, and hence, require raw materials and energy. Depending on the production method, emissions and pollution will also be produced, and in most cases, they are nonbiodegradable and are difficult to recycle. Thus, demonstrating a negative impact on the environment. 

#### 4.2.1. Polymeric Fibres

Polypropylene fibres have excellent mechanical properties and are one of the most commonly used and appreciated fibres. Their advantages include being noncorrosive [4] and their resistance to alkali [8,9,11], chemicals [4], and chlorides [4], thus making them a suitable reinforcement fibre for a diverse range of applications. A disadvantage of polypropylene fibres is that they have a relatively low density, which causes floating issues in some composite matrices [9]. They also possess low hydrophilic characteristics [9] as they are hydrophobic [4], which could affect their bonding with certain matrices [9].

For high-density polyethylene, certain limitations in terms of applications arise, as polyethylene has low tensile strength [9].

Polyethylene terephthalate (PET) fibres have the highest density of the three, and they have good mechanical properties, such as tensile strength and elastic modulus, and can mix easily in matrices such as concrete [9]. A disadvantage of PET fibres is that they take a lot more energy to produce due to the production method, hence these fibres are more costly. Another disadvantage of PET fibres is that they show degradation in alkali environments [9,11], thus limiting their use in a range of applications.

Some limitations of polymeric fibres include their low biodegradability, and capabilities for recycling and re-use. When recycling plastic, there is a risk if the prior history of the plastic is not known. If it was procured from an uncontrolled environment, the resulting properties of the recycled fibre may be unstable and inconsistent [9].

#### 4.2.2. Steel Fibres

Steel fibres have shown many advantages when used as reinforcement for composite materials. Research has shown that when steel fibres are used to reinforce concrete structures, there are many improvements in the properties [8]. Steel fibres help improve the behaviour of concrete in respect of cracking, shrinkage [9], ductility, toughness, resistance to fatigue, impact, and blast loading [8]. Furthermore, strength properties, such as tensile strength, compressive strength, and flexural strength and capacity are increased for the parent material [6,8,9,10,22]. Another advantage of steel fibres is that they can be an ideal additive to specific applications as they possess good electric, magnetic, and heat conductivity [9].

The disadvantages and limitations of steel fibres are comparable with those of steel reinforcement. They are quite vulnerable to corrosion [9], which may lead to degradation and deterioration of the parent material [9]. Compared to other fibres, a larger quantity for steel fibres may be required. This was demonstrated in a study where 7 and 40 kg/m^3^ quantities of synthetic barchip and steel fibres were used, respectively, to reinforce concrete slabs, and the results demonstrated that the synthetic fibres performed similarly to the steel fibres [37].

#### 4.2.3. Glass Fibres

Glass fibres have great mechanical properties and excel in terms of strength [3,9,25,26], toughness, stiffness, thermal properties [3,25], durability [3], performing well in high temperature environments [9,25], and in interfacial bonding with the matrix [3]. Glass fibres are most frequently used as reinforcement in resins and composites [3] as they have amazing properties in strengthening composites [9]. Glass fibre composites have excellent properties when considered and designed carefully [19].

Although there are many advantages and benefits to using glass fibres, there are disadvantages as the production of glass fibres leads to concerns for the environment [3] and questionable sustainability. Manufacturing requires quantities of B_2_O_3_ (boric acid), which may not be sustainable as the compound is limited [3]. Glass fibres have a higher density, require more energy to fabricate, have lower recycling potential, and may be a potential hazard when in particle form [60]. For example, the total energy used to make a flax fibre mat only requires about 17% of the energy required to make a glass fibre mat [60].

The problem of disposal for glass products and glass fibre composites during the end of life phase is also well known [3,19]. For most glass fibre composites, recycling could be a possibility, however most products are simply burnt or buried [19], which is not good for the environment. Another disadvantage of glass is that it degrades in alkaline environments, as it has poor alkali resistance [9]. This limits them in terms of concrete applications, as concrete has an alkaline environment.

#### 4.2.4. Carbon Fibres

Carbon fibres have been added in materials to form composites with improved properties [3]. The addition of carbon fibres creates a composite that has outstanding mechanical properties [3], performs well in high temperature environments [3,25], and possesses the benefit of durability [3]. A disadvantage of carbon fibres is that due to their great properties it is highly expensive to manufacture carbon fibres [3,29]. The bonding between the fibres and the material matrix may also present a challenge [3].

The production of carbon fibres also leads to concern for the environment [3] and questionable sustainability. The problem of the disposal of carbon fibre composites during the end of life phase is also well known [3,19]. For most carbon fibre composites, recycling could be a possibility, however most products are simply burnt or buried [19], which is not good for the environment.

### 4.3. Basalt Fibres

Basalt fibres have many advantages, as they have very good physical and mechanical properties, making them competitive contenders as reinforcement for composites [3,19,29]. Basalt fibres are completely natural and have been labelled as a green industrial material [3]. Basalt fibres show significant environmental benefits, as the recycling at their end of life is much more efficient than that of other fibres [3]. The melting point of basalt is very high; hence, when a composite material containing basalt fibre is recycled, the basalt in the form of powder is left over from the process and obtained. This powder still has great value and can be reused in various applications [3]. Basalt products are safe [30]. In addition, they are nontoxic, noncancerous, do not react with air or water, are noncombustible, fire proof, and do not have harmful reactions with chemicals [3,30].

The raw material is readily available and during the manufacturing procedure absolutely nothing is added to the molten basalt [3]. This means that the fibre is made solely from the basalt itself and is completely natural [31]. The fibres show great potential in respect to sustainability and being environmentally friendly.

Although there are many benefits when using basalt fibre, there is a disadvantage. It has been found that, independently, basalt fibres do not perform well in alkali environments [7]. This may limit their applications, however, basalt fibres have been successfully used in alkali environments before, although alteration or certain additives may be required.

### 4.4. Waste Materials and Fibres

Waste materials contain fibres that could potentially be utilised in applications as a cost effective, environmentally friendly, and sustainable option [32]. There are many advantages of utilising waste materials and fibres in construction materials. The volume of waste that ends up in landfills can be greatly reduced; the energy, resources and extraction processes that are required when procuring virgin materials [33] can be conserved; and the energy used for the combustion process of landfill materials can also be saved. There will also be a reduction in terms of the pollution and emissions, which is good for the environment.

Various waste materials, such as tyres, carpet, natural hair, coconut fibre, glass, steel slag, plastic, and cigarette butts, were discussed in this review. Through various studies, it was determined that there were benefits in utilising waste materials in construction materials as a sustainable and cost-effective solution for some applications.

In SMA mixtures, waste tyre and carpet materials increased the toughness and tensile strength, stopped down drain from occurring, and provided stability to the mixture. It provides a cost effect alternative and demonstrates sustainable value [32]. Previous experiments and studies have affirmed that when crumb rubber was recycled in the concrete slab, the slab had improved resistance to fire and the spalling impairments created by the fire was reduced [40]. Waste carpet fibres have been used to reinforce clay soils as they have increased the shear strength [45,51], the effective cohesion [45], the deviator stress [51], effective stress ratio [51], the unconfined compression strength (UCS) [52], and reduced swelling [47]. Waste carpet fibres have also been successfully used to enhance the bearing resistance properties of slopes [49].

Natural hair was investigated, and it improved the strength and stability of the clay soil. The optimum fibre content was 2%. It was suggested that hair could possibly be used to reinforce embankments and help stabilise slopes [34]. Coconut fibres have been used to reinforce and increase the strength of soil, where the optimal fibre content was 0.75% [4]. Steel slag showed the potential to be a great substitute for coarse aggregates in surface asphalt [33].

Lastly, it was determined that cigarette butts could successfully be utilised in fired clay bricks and in asphalt concrete when encapsulated [35,40]. These studies show how to dispose and make use of toxic waste materials.

The disadvantages of using waste materials depend on what application they are being used for. In asphalt pavements there are concerns over the potential effects, such as run-off pollutants and leaching [33]. Concerns also arise when using industrial waste, such as steel slag, due to such properties as electric conductivity and concentration of chromium [33]. The processing cost for recycling and utilising the waste materials may often be higher than the typical cost of virgin materials, which limits their appeal for usage [33].

### 4.5. Important Factors to Consider when Using Fibres

Throughout various research and studies, it has been emphasised that the content of fibres used to reinforce the composite material is very important. A higher fibre content is not necessarily better. Although fibre reinforcement is known to improve the mechanical properties of materials, this is usually only up to a certain extent, and, if exceeded, the properties will start to decrease. This is why the optimal fibre content within a composite material is important, as the best results can be obtained. Physical properties, such as the fibre length, the fibre count, and the distribution of fibre, should be considered [12]. In concrete, as the quantity of fibres increases, the workability of the concrete mixture decreases, hence additives may be required [8,9,11]. In addition, in soil short fibres were reported to be more effective [4]. Treatment may also be used to improve the properties of fibres such as water repellence, alkaline treatment, and surface improvement [2].

Fibres have been used to develop high performing composites with other matrices [26]. The physicochemical interaction between the components of the composite, the degree of adhesion, and the bonding between the fibres and the matrix are extremely important factors that influence the mechanical behaviour of the composite [26,29,59]. The finest composite properties are typically the result of a strong interface between the fibres and the matrix [25]. Additives, such as coupling agents, have been used to improve the adhesion between the composites to create stronger bonds. They help bond and unite the fibres with the matrix [25], hence creating a composite with the best properties.

## 5. Conclusions

In this study, the type, properties, and applications of different fibres used in a wide range of construction materials including normal concrete, asphalt concrete, soil, earth materials, blocks and bricks, composites, and other applications were reviewed. Their advantages, disadvantages, and limitations were also discussed.

Natural fibres are readily available, energy efficient, low-cost, economical, can reduce the environmental impact, and save raw materials and energy. They are biodegradable, renewable, and lightweight. Natural fibres could potentially replace synthetic fibres for some applications, however, natural fibres have poor durability and degrade over time.

Synthetic fibres can reduce the cost of construction as they can be used to substitute traditional reinforcement, such as steel mesh and steel rebars, which are much heavier and require more energy, resources, and time to produce. However, synthetic fibres are man-made, require raw materials and energy, are difficult to recycle, and demonstrate negative environmental impact.

Basalt fibres are natural, safe, nontoxic, and noncancerous fibres that have very good physical and mechanical properties, thereby making them competitive contenders as reinforcement. The raw material is readily available and during the manufacturing procedure absolutely nothing is added to the molten basalt. Basalt fibres show significant environmental benefit, as the recycling at its end of life is much more efficient than that of synthetic fibres.

The build-up of waste materials all around the world is a known issue as landfill space is limited and the incineration process requires a lot of energy and produces unwanted emissions. Through the research of various studies, the utilisation of waste materials in construction materials has shown great potential. Although some properties may decrease when the waste fibres are added, the slightly lower properties compared to the cost and energy saved could be a sustainable and environmentally beneficial trade off. In some cases, the processing cost for recycling and utilising the waste materials may be higher than the typical cost of virgin materials, which limits their appeal for usage.

## 6. Recommendations

For natural fibres, further study concerning the improvement of long-term durability and the long-term effect of the techniques used is recommended. For synthetic fibres, further study is suggested in respect to the development of a biodegradable polymer that is reinforced by a recyclable fibre, to form a composite material that is more sustainable and environmentally friendly. Lastly, for waste materials, further study concerning the long-term effects of waste fibres and the use of other kinds of waste fibres in construction materials is recommended.

## Figures and Tables

**Table 1:** Differentiating micro synthetic fibres with macro synthetic fibres. (Adapted from Yin et al. 2015, [9]).

Geometry	Micro Synthetic Fibre	Macro Synthetic Fibre
Diameter/cross-section	5–100 µm	0.6–1 mm^2^
Length	5–30 mm	30–60 mm

**Table 2:** Classification of basalt. (Adapted form Soares et al. 2016 [19]).

Classification	Content of Silicon Dioxide
Alkaline basalt	SiO_2_ < 42%
Mildly acidic basalt	42% ≤ SiO_2_ < 46%
Acidic basalt	SiO_2_ ≥ 46%

**Table 3:** Difference between short and continuous basalt fibre. (Adapted from Hafsa & Rajesh, 2015 and Deák & Czigány, 2009 [3,28]).

Differences	Short Basalt Fibre	Continuous Basalt Fibre
Production Method	Melt Blowing/Junkers Method	Spinneret Method
Quality	Lower	Higher
Properties	Weaker	Stronger
Deviances in Properties	Uneven	Uniform
Cost	Lower	Higher

## Appendix A

**Table A1:** Summary table of fibres.

Type of Fibre	Physical Properties		Mechanical Properties		Reference
**Natural Fibres**					
***Leaf fibres***					
Pineapple fibre	Length:	1 cm	n/a	n/a	[2]
Sisal fibre	Average length:	862 mm (long)	n/a	n/a	[5]
		661 mm (medium)			
		543 mm (short)			
	Average diameter:	0.15 mm			
	Natural humidity:	13.3%			
	Specific weight:	9.3 kN/m^3^			
Sisal fibre	Length:	10, 15, 20, 25 mm Optimal was 20 mm	Ultimate tensile strength: Modulus of elasticity:	560 MPa 26–32 GPa	[4]
	Diameter:	25–400 µm			
	Specific gravity:	1.2–1.45 g/cm^3^			
Sisal fibre	Length:	4–160 cm	Tensile strength: Modulus of elasticity:	233–580 MPa 5–18 GPa	[2]
	Diameter:	0.004–0.3 mm			
	Absolute density:	1370 kg m^−3^			
	Absorption:	110%			
Banana fibre	Length:	0.85–1.7 cm	n/a	n/a	[2]
***Palm tree fibres***					
Coconut (coir) fibre	Average Length:	186 mm (long)	n/a	n/a	[5]
		105 mm (short)			
	Average diameter:	0.27 mm			
	Natural humidity:	13.7%			
	Specific weight:	9.5 kN/m^3^			
Coconut (coir) fibre	Length:	10–500 mm and 50 mm	Ultimate tensile strength:	250 MPa	[4]
	Diameter:	10–20 µm	Modulus of elasticity:	4–5 GPa	
	Specific gravity:	1.15–1.33 g/cm^3^			
Coconut (coir) fibre	Length:	3.5–5 cm	Tensile strength:	73–505 MPa	[2]
	Diameter:	0.27–2.38 mm	Modulus of elasticity:	3–40 GPa	
	Absolute density:	1177 kg m^−3^			
	Absorption:	100%–145%			
Date palm fibre	Length:	2.5–3.5 cm	Tensile strength:	233 MPa	[2]
	Diameter:	0.1–0.8 mm	Modulus of elasticity:	5 GPa	
	Apparent density	512–1089 kg m^−3^			
	Absolute density:	1300–1350 kg m^−3^			
	Absorption:	97%–203%			
Date palm fibre	Diameter:	20 mm	Tensile strength:	290 ± 20 MPa	[54]
		60 mm		240 ± 30 MPa	
		100 mm		170 ± 40 MPa	
	Apparent specific weight:	512.21–1088.81 (kg/m^3^)	Elongation at break:	0.23%	
	Absolute specific weight:	1300–1450 (kg/m^3^)			
	Water absorption (24 hrs):	96.83%–202.64%			
	Humidity:	9.5%–10.5%			
Palm fibre (unknown type)	Length:	15, 20, 30, 40, 45	Ultimate tensile strength:	31–60 MPa	[4]
		Optimal was 30 mm	Modulus of elasticity:	0.55 GPa	
	Diameter:	25–60 µm			
	Specific gravity:	1.3–1.46 g/cm^3^			
Oil palm fibre	Average length:	25 mm	Tensile strength:	35.33 MPa	[55]
	Average diameter:	19.4 µm			
	Specific gravity:	2.13			
	Water absorption (%):	0.79			
***Bast fibres***					
Jute fibre	Length:	5, 10, 14, 20	Ultimate tensile strength:	453–550 MPa	[4]
		Optimal was 10 mm	Modulus of elasticity:	22 GPa	
	Diameter:	10–50 µm			
	Specific gravity:	1.44–1.46 g/cm^3^			
Jute Fibre	Length:	2–4 cm	Tensile strength:	400–800 MPa	[2]
	Diameter:	1 mm	Modulus of elasticity:	10–30 GPa	
	Absolute density:	1700 kg m^−3^			
Hemp fibre	Length:	0.85–1.7 cm	Tensile strength:	900–1077 Mpa	[2]
	Diameter:	0.0035 mm	Modulus of elasticity:	21–34 GPa	
	Absolute density:	1500 kg m^−3^			
	Absorption:	80%–105%			
	Thermal conductivity:	41.4–48.6 mWm^−1^ K^−1^			
Hemp fibre	Absolute density:	1460 kg/m^3^	n/a	n/a	[17]
Hemp hurds	Length:	0.5–3.5 cm	n/a	n/a	[2]
	Diameter:	1–8 mm			
	Absorption:	280%			
Flax fibre	Length:	7–8.5 cm	Tensile strength:	805 MPa	[2]
	Diameter:	0.035 mm	Modulus of elasticity:	21 GPa	
Kenaf Fibre	Length:	3 cm	Tensile strength:	1000 Mpa	[2]
	Diameter:	0.13 mm	Modulus of elasticity:	136 GPa	
	Absolute density:	1040 kg m^−3^			
	Absorption:	307%			
Diss fibre	Length:	2 cm	Tensile strength:	100 Mpa	[2]
***Cereal straw fibres***					
Barley straw fibre	Length:	10–500 mm	n/a	n/a	[4]
	Diameter:	1000–4000 µm			
	Specific gravity:	2.05 g/cm^3^			
Barley straw fibre	Length:	0.5–6 cm	n/a	n/a	[2]
	Diameter:	1–4 mm			
	Apparent density:	47–106.9 kg m^−3^			
	Absolute density:	870–2050 kg m^−3^			
	Absorption:	400%–600%			
Barley straw fibre:	Length:	5–20 mm	n/a	n/a	[17]
	Bulk density:	47 kg/m^3^			
	Absolute density:	870 kg/m^3^			
	Porosity:	94%			
Wheat straw fibre	Length:	0–5 cm	n/a	n/a	[2]
	Diameter:	0.5–3 mm			
	Apparent density:	33–103.6 kg m^−3^			
	Absolute density:	868 kg m^−3^			
	Absorption:	230%–350%			
	Thermal conductivity:	41.4–48.6 mWm^−1^ K^−1^			
Wheat straw fibre	Length:	30 mm	n/a	n/a	[17]
	Bulk density:	25–33 kg/m^3^			
	Absolute density:	865–871 kg/m^3^			
	Porosity:	96%–97%			
Pavement straw composite fibre	Length:	1 ± 0.1 mm	Tensile strength:	520 MPa	[41]
	Oil absorption:	5.78	Fracture elongation:	12%	
	Melting temperature:	160 °C			
***Cellulose fibres***					
Lignin fibre	Average length:	1.1 mm	n/a	n/a	[44]
	Average diameter:	0.045 mm			
	Length/diameter ratio (mean):	24			
	Specific surface area:	118.1 (10^−3^ m^2^/g)			
	Specific gravity:	0.8–1.3			
	Melting temperature:	200 °C			
	
Lignocellulose fibre	Length:	0.21 mm	Tensile strength:	475 MPa	[41]
	Oil absorption:	5.16	Fracture elongation:	10%	
	Melting temperature:	160 °C			
***Hair fibres***					
Human hair fibre	Length:	25 mm	Tensile strength:	400 MPa	[34]
	Diameter:	50 µm	Flexural strength:	25–30 MPa	
	Cross-section shape:	Circular	Elongation:	150% of the dry weight	
	Linear density:	1.25–1.40 gm/cc			
Sheel wool	Length:	1, 2, 3 cm	n/a	n/a	[2]
	Diameter:	0.035 mm			
***Wood fibres***					
Wood (fibre/shavings)	Length:	0.3–2 cm	n/a	n/a	[2]
	Diameter:	0.025–0.05 mm			
	Apparent density:	50–114.4 kg m^−3^			
	Absolute density:	440 kg m^−3^			
	Absorption:	240%			
	Thermal conductivity:	35.3–53.9 mWm^−1^K^−1^			
***Synthetic Fibres***					
***Plastic fibres***					
Polymeric fibre (Polypropylene and polyethylene composite macro fibre)	Average length:	40 mm	Tensile strength:	600 MPa	[6,10]
	Aspect ratio:	90		620 MPa [6]	
	Specific gravity:	0.92	Modulus of elasticity:	9.5 GPa	
	Cross-section shape:	Rectangular			
	Average thickness:	0.105 mm			
	Average width:	1.4 mm			
PET fibre (recycled bottle)	Average length:	20 mm	n/a	n/a	[11]
	Average diameter:	25–30 µm			
	Density:	1.33 g.cm^−3^			
	Water absorption:	3.30%			
	Vitreous Temperature:	62 °C			
	Fusion Temperature:	252.8 °C			
PET fibre (recycled bottle)	Density:	1.38 g/cm^3^	Tensile strength:	420 MPa	[9]
				450 MPa	
				160 MPa	
				10 GPa	
				3 GPa	

			Modulus of elasticity:	(dependent upon manufacturing method and condition of the recycled bottles)	
Polypropylene fibres (Barchip, shogun)	Average length:	48 mm	Tensile strength:	640 MPa	[37]
	Specific gravity:	0.9–0.92	Modulus of elasticity:	10 GPa	
	Texture of surface:	Embossed continuously			
	Number of fibres per kg:	>59,500			
	Melting point:	159–179 °C			
	Ignition point:	>450 °C			
	Base Resin:	Olefin (modified)			
High Performance Polypropylene fibres (Barchip)	Average length:	48 mm	Tensile strength:	550 MPa	[8]
	Specific gravity:	0.9–0.92	Modulus of elasticity:	10 GPa	
	Texture of surface:	Embossed continuously			
	Number of fibres per kg:	>35,000			
	Melting point:	150–165 °C			
	Ignition point:	>450 °C			
	Base Resin:	Polyelifen			
Polypropylene fibre	Length:	5, 12, 18, 24, 35, 50 mm	Ultimate tensile strength:	120–450 MPa	[4]
	Diameter:	23–150 µm	Modulus of elasticity:	3–3.5 GPa	
	Specific gravity:	0.92 g/cm^3^			
Polypropylene fibre	Length:	8 mm	Tensile strength:	585.6 MPa	[41]
	Oil absorption:	3.17	Fracture elongation:	24.30%	
	Melting temperature:	166 °C			
Polyester fibre	Average length:	6.35 mm	Tensile strength:	517 ± 34.5 MPa	[44]
	Average diameter:	0.02 mm			
	Length/diameter ratio (mean):	318			
	Specific surface area:	20.1 (10^−3^ m^2^/g)			
	Specific gravity:	1.36 ± 0.04			
	Ductility:	33% ± 9%			
	Melting temperature:	>249 °C			
Polyester fibre	Average length:	6.00 mm	Tensile strength:	531 MPa	[44]
	Average diameter:	0.02 mm			
	Length/diameter ratio (mean):	300			
	Specific surface area:	14.8 (10^−3^ m^2^/g)			
	Specific gravity:	1.36 ± 0.04			
	Ductility:	>50%			
	Melting temperature:	>249 °C			
Polyester fibre	Length:	3, 6, 12, 20, 64	Ultimate tensile strength:	400–600 MPa	[4]
	Diameter:	30–40 µm	Modulus of elasticity:	10–30 GPa	
	Specific gravity:	1.35 g/cm^3^			
Polyester fibre	Length:	6 mm	Tensile strength:	840 MPa	[41]
	Oil absorption:	2.85	Fracture elongation:	28%	
	Melting temperature:	251 °C			
Polyethylene fibre	Length:	12, 25, 50 mm	Ultimate tensile strength:	100–620 MPa	[4]
	Diameter:	400–800 µm	Modulus of elasticity:	0.14–1 GPa	
	Specific gravity:	0.92 g/cm^3^			
Polyvinyl alcohol fibres	Length:	12 mm	Ultimate tensile strength:	1078 MPa	[4]
	Diameter:	100 µm	Modulus of elasticity:	25 GPa	
	Specific gravity:	1.3 g/cm^3^			
Polyacrylonitrile fibre	Average length:	4–6 mm	Tensile strength:	>910 MPa	[44]
	Average diameter:	0.02 mm			
	Length/diameter ratio (mean):	385			
	Specific surface area:	22.3 (10^−3^ m^2^/g)			
	Specific gravity:	1.14–1.16			
	Ductility:	8%–12%			
	Melting temperature:	>240 °C			
***Steel fibres***					
Steel Fibre	Average length:	32 mm	Tensile strength:	>1000 MPa	[8]
	Average diameter:	0.6 mm			
	Specific gravity:	7.85			
Steel fibre	Average length:	60% ± 5% mm	Tensile strength:	1035 MPa	[42]
	Maximum diameter:	0.75% ± 5% mm	Modulus of elasticity:	200,000 MPa	
	Slenderness ratio:	90			
	Number of fibres per kg:	4,600			
Steel Fibre (Hooked end)	Average length:	60 mm	Tensile strength:	1000 MPa	[6]
	Average diameter:	0.92 mm	Modulus of elasticity:	180 GPa	
	Aspect Ratio:	0.65			
	Specific gravity:	7.8			
	Cross-section shape:	Circular			
Steel Fibre (Crimped)	Average length:	50 mm	Tensile strength:	1000 MPa	[6]
	Average diameter:	1.3 mm	Modulus of elasticity:	180 GPa	
	Aspect ratio:	40 mm			
	Specific gravity:	7.8			
	Cross-section shape:	Circular			
***Glass Fibres***					
Glass fibre	Length:	25 mm	Ultimate tensile strength:	1500–5000 MPa	[4]
	Diameter:	3–19 µm	Modulus of elasticity:	53–95 GPa	
	Specific gravity:	2.49–2.60 g/cm^3^			
Glass fibre	Density:	2.55 g/cm^3^	Tensile strength:	1750 MPa	[26]
			Tensile modulus:	70 GPa	
Glass fibre (S-2)	Diameter:	12.3 µm	Tensile strength:	2295 MPa	[31]
	Density:	2.52 g/cm^3^	Tensile Modulus:	78.3 GPa	
	Water absorption:	0.80%	Elongation at break:	3.35%	
Glass Fibre (E-glass)	Average Diameter:	16.8 ± 1.6 µm	Tensile strength:	1472 ± 395 MPa	[28]
	Density:	2.61 g/cm^3^	Modulus of elasticity:	57.0 ± 3.0 GPa	
	Area or cross-section:	223.4 ± 42 µm^2^	Maximum force:	0.32 ± 0.08 N	
			Extension at failure:	0.68 ± 0.22 mm	
			Breaking strain:	2.71% ± 0.86%	
Glass Fibre (E-glass)	Diameter:	6–21 µm	Tensile strength:	3100–3800 MPa	[3]
	Density:	2.54–2.57 g/cm^3^	Modulus of elasticity:	72.5–75.5 GPa	
	Specific gravity:	2.5–2.62	Elongation at break:	4.70%	
	Sound absorption coefficient:	0.8–0.93			
Glass Fibre (S2 glass)	Diameter:	6–21 µm	Tensile strength:	4020–4650 MPa	[3]
	Density:	2.54 g/cm^3^	Modulus of elasticity:	83–97 GPa	
	Specific gravity:	2.46	Elongation at break:	5.30%	
Glass fibre (E-glass)	Density:	2.56 g/cm^3^	Tensile strength:	1.4–2.5 GPa	[30]
			Modulus of elasticity:	76 GPa	
			Elongation to fracture:	1.8%–3.2%	
			Specific E modulus	30 GPa per g/cm^3^	
			Specific tensile strength:	0.5–1 GPa per g/cm^3^	
Crushed waste glass	Bulk density:	1360 kg/m^3^	Los Angeles Value:	38.4, 24.8–27.7, 27.7	[24]
	Specific gravity:	2.4–2.8, 2.5, 2.52			
	Shape index:	30.50%	CBR:	50%–75%	
	Fineness modulus:	4.25, 0.43–3.29	Friction angle:	Crit = 38 (loose RG)	
	Flakiness index:	84.4–94.7	Friction angle:	80–61 (dense RG)	
***Carbon Fibre***					
Carbon fibre	Diameter:	5–15 µm	Tensile strength:	3500–6000 MPa	[3]
	Density:	1.78 g/cm^3^	Modulus of elasticity:	230–600 GPa	
	Specific gravity:	1.75–1.95	Elongation at break:	1.5%–2.0%	
Carbon nano-fibres	Diameter:	60–150 nm	Tensile strength:	7 GPa	[22]
	Length:	30–100 µm	Modulus of elasticity:	400 GPa	
***Mineral Fibres***					
Asbestos	Average length:	5.5 mm	Tensile strength:	30–40 MPa	[44]
	Specific surface area:	60 (10^−3^ m^2^/g)			
	Specific gravity:	2.75			
Short Basalt Fibre	Average Diameter:	10 ± 3.1 µm	Tensile strength:	602 ± 295 MPa	[28]
	Density:	2.66 g/cm^3^	Modulus of elasticity:	48.2 ± 20.6 GPa	
	Area or cross-section:	90.2 ± 56.7 µm^2^	Maximum force:	0.05 ± 0.04 N	
			Extension at failure:	0.32 ± 0.09 mm	
			Breaking strain:	1.29% ± 0.48%	
Continuous basalt fibre	Average Diameter:	14.2 ± 1.4 µm	Tensile strength:	2016 ± 434 MPa	[28]
	Density:	2.56 g/cm^3^	Modulus of elasticity:	61.9 ± 3.5 GPa	
	Area or cross-section:	160.2 ± 30.3 µm^2^	Maximum force:	0.32 ± 0.09 N	
	Colour:	Grey to dark green	Extension at failure:	0.89 ± 0.22 mm	
			Breaking strain:	3.56% ± 0.89%	
Continuous basalt fibre	Average Diameter:	12.7 ± 1.5 µm	Tensile strength:	1608 ± 350 MPa	[28]
	Density:	2.64 g/cm^3^	Modulus of elasticity:	62.0 ± 3.6 GPa	
	Area or cross-section:	90.2 ± 56.7 µm^2^	Maximum force:	0.21 ± 0.07 N	
			Extension at failure:	0.68 ± 0.17 mm	
			Breaking strain:	3.47% ± 0.7%	
Continuous basalt fibre	Average Diameter:	14.1 ± 2.9 µm	Tensile strength:	1811 ± 331 MPa	[28]
	Density:	2.63 g/cm^3^	Modulus of elasticity:	53.2 ± 7.4 GPa	
	Area or cross-section:	163.5 ± 63.3 µm^2^	Maximum force:	0.3 ± 0.13 N	
			Extension at failure:	0.87 ± 0.18 mm	
			Breaking strain:	3.47% ± 0.7%	
Basalt Fibre	Density:	2.6–2.7 g/cm^3^	n/a		[28]
	Melting temperature:	1350–1700 °C			
	Temperature range for use:	−200 to + 600 °C			
Basalt Fibre	Diameter:	6–21 µm	Tensile strength:	3000–4000 MPa	[3]
	Density:	2.63–2.8 g/cm^3^	Modulus of elasticity:	93–110 GPa	
	Specific gravity:	2.65–2.8	Elongation at break:	3.1%–6%	
	Sound absorption coefficient:	0.95–0.99			
	Melting temperature:	1450 °C			
		1400 °C			
Basalt Fibre	Density:	220 g/m^2^	Modulus of elasticity:	85 GPa	[19]
	Melting temperature:	1500 °C			
Basalt Fibre	Melting temperature:	1447 °C	n/a	n/a	[29]
	Maximum application temperature:	982 °C			
	Sustained operating temperature:	820 °C			
	Minimal operating temperature	−258 °C			
Basalt fibre	Density:	2.8 g/cm^3^	Tensile strength:	2.8 GPa	[30]
	Temperature range for use:	−200 to + 800 °C	Modulus of elasticity:	89 GPa	
	Melting temperature:	1500–1700 °C	Elongation to fracture:	3.15%	
			Specific E modulus	31.78 GPa per g/cm^3^	
			Specific tensile strength:	1 GPa per g/cm^3^	
Basalt fibre	Diameter:	10.2 µm	Tensile strength:	1828 MPa	[31]
	Density:	2.63 g/cm^3^	Tensile modulus:	72.2 GPa	
	Water absorption:	0.15%	Elongation at break:	3.03%	
***Waste fibres***					
Carpet waste fibres (Mainly propylene fibres, styrene-butadiene rubber, nylon and wool)	Length:	2 to 20 mm	Specific tensile modulus:	0.27–0.7 GPa/g/cm^3^	[51]
	Thickness:	80 to 1500 µm			
	Specific gravity:	0.9–1.32			
	Water absorption:	0%–4.5%			
Carpet waste fibres (short nylon fibres)	Length:	2 to 20 mm	Specific tensile modulus:	0.4–0.7 GPa/g/cm^3^	[51]
	Thickness:	80 to 1500 µm			
	Specific gravity:	1.14			
	Water absorption:	4.1%–4.5%			
